# Toward Learning in Neuromorphic Circuits Based on Quantum Phase Slip Junctions

**DOI:** 10.3389/fnins.2021.765883

**Published:** 2021-11-08

**Authors:** Ran Cheng, Uday S. Goteti, Harrison Walker, Keith M. Krause, Luke Oeding, Michael C. Hamilton

**Affiliations:** ^1^Department of Electrical and Computer Engineering, Auburn University, Auburn, AL, United States; ^2^Alabama Micro/Nano Science and Technology Center, Auburn University, Auburn, AL, United States; ^3^Department of Physics, University of California, San Diego, San Diego, CA, United States; ^4^Department of Materials Engineering, Auburn University, Auburn, AL, United States; ^5^Department of Mathematics and Statistics, Auburn University, Auburn, AL, United States

**Keywords:** quantum phase slip junction, Josephson junction, neuromorphic computing, spike timing dependent plasticity, unsupervised learning, coupled synapse networks

## Abstract

We explore the use of superconducting quantum phase slip junctions (QPSJs), an electromagnetic dual to Josephson Junctions (JJs), in neuromorphic circuits. These small circuits could serve as the building blocks of neuromorphic circuits for machine learning applications because they exhibit desirable properties such as inherent ultra-low energy per operation, high speed, dense integration, negligible loss, and natural spiking responses. In addition, they have a relatively straight-forward micro/nano fabrication, which shows promise for implementation of an enormous number of lossless interconnections that are required to realize complex neuromorphic systems. We simulate QPSJ-only, as well as hybrid QPSJ + JJ circuits for application in neuromorphic circuits including artificial synapses and neurons, as well as fan-in and fan-out circuits. We also design and simulate learning circuits, where a simplified spike timing dependent plasticity rule is realized to provide potential learning mechanisms. We also take an alternative approach, which shows potential to overcome some of the expected challenges of QPSJ-based neuromorphic circuits, via QPSJ-based charge islands coupled together to generate non-linear charge dynamics that result in a large number of programmable weights or non-volatile memory states. Notably, we show that these weights are a function of the timing and frequency of the input spiking signals and can be programmed using a small number of DC voltage bias signals, therefore exhibiting spike-timing and rate dependent plasticity, which are mechanisms to realize learning in neuromorphic circuits.

## 1. Introduction

Neuromorphic computing has been a rich area of study over the past several decades, bringing together the fields of electronics, biology, materials and computer science, among others (Mead, 1990). A Von Neumann (or Princeton) architecture (Burks et al., 1982), as well as the closely-related Harvard architecture, have been the basis of most computational systems since their conception. These architectures employ a central processing unit that works alongside a dedicated memory that stores data and instructions together for von Neumann architectures or, in the case of the Harvard architecture, separately. The processor and memory must communicate with each other to process information, requiring movement of data and instructions, leading to an information flow bottleneck that provides one limitation for the speed of computation. Neuromorphic hardware attempts to mimic a biological brain, specifically a human brain, and is organized with both processing and memory distributed among the system, with a goal of reducing the inherent latency found in von Neumann-like systems. Though our current understanding of the brain is most certainly not a complete one, efforts to mimic nature are expected to lead us to more efficient computational architectures and a deeper understanding of the human brain. It has been claimed that efficient emulation of scalable biological neural networks could allow for computation that negates the information bottleneck associated with von Neumann like architectures and provides a low power platform more apt for neural networks and parallel processing (Monroe, 2014). Different approaches have been taken to realize physical electronics hardware for neuromorphic circuits that imitate some of the useful functions of the brain, primarily including semiconductor-based electronics such as complementary metal oxide semiconductor (CMOS) approaches (Mead, 1990; Seo et al., 2011; Merolla et al., 2014), memristive devices (Jo et al., 2010; Sung et al., 2018) and organic electronics (van de Burgt et al., 2017; Pecqueur et al., 2018). While the performance and scale of some of these systems is impressive, reaching the extremely low power consumption (or energy per operation) and the massive level of interconnection of the human brain still remain big challenges. When compared to semiconducting devices, superconductive devices demonstrate drastically lower, nearly zero, power dissipation and are competitive even when considering the necessary cooling to cryogenic temperatures (Holmes et al., 2013). Superconductive circuit elements, such as Josephson junctions (JJs), magnetic JJs (MJJs), superconducting nanowire single photon detectors (SNSPDs), and quantum phase slip junctions (QPSJs), have been shown to compete with the ultralow power consumption of the brain (Crotty et al., 2010; Schneider et al., 2018a,b). Superconductive electronics (SCE), with lossless superconducting interconnects, can also allow the massive interconnections needed to realize complex neuromorphic systems. Furthermore, the non-linear switching dynamics of superconductive devices allow realization of spiking behavior with non-volatile memory in the form of spike timing dependent plasticity (STDP), which is a biologically plausible learning mechanism. With these benefits in mind, we are exploring superconductive electronics based circuits to create a scalable system of neurons and synapses that can be integrated to form learning circuits.

One-dimensional (or quasi-one-dimensional) (1D) superconductivity has been an active subject of research due to resultant interesting physical effects. In a superconducting 1D nanowire, quantum phase slip (QPS) causes the wire to demonstrate an insulating, zero-current state when an applied voltage is below a critical value and to exhibit resistive behavior when above (Mooij and Harmans, 2005). This leads to a measurable resistive tail at temperatures significantly below the superconducting critical temperature (Giordano, 1988), or as a single-electron charging effect in nano-scale tunnel junctions (Fulton and Dolan, 1987). Quantum phase slip occurs along with coherent tunneling of fluxons across superconducting nanowires. The phase difference along the wire, along the phase slip region, changes by 2π and the superconducting order parameter is reduced to zero within the phase slip region (Kerman, 2013). This tunneling of magnetic flux through the superconducting nanowire has been identified as a quantum dual to Josephson tunneling of Cooper pairs across an insulating charge tunnel barrier (Mooij and Nazarov, 2006). Several experiments have been conducted over the past few years to demonstrate coherent quantum phase slip behavior in superconducting nanowires (Astafiev et al., 2012; Webster et al., 2013; Constantino et al., 2018). These phase slip events have been suggested for applications such as a quantum current standard (Wang et al., 2019), single charge (Hongisto and Zorin, 2012) and single flux transistors (Kafanov and Chtchelkatchev, 2013), superconducting qubits (Mooij and Harmans, 2005), and digital computing (Goteti and Hamilton, 2018; Hamilton and Goteti, 2018). In addition to these suggested applications, the stochastic nature of occurrence of coherent quantum phase-slips in nanowires can be particularly applicable for neuromorphic computing. Recently, there have been promising results for an algorithm-level, digit recognition approach using models for QPSJ-based superconductive circuitry, which furthermore shows the growing interest in this area (Zhang et al., 2021).

Quantum phase slip junctions (QPSJs) are promising superconductive electronic devices for applications in high-speed and low-power neuromorphic computing (Cheng et al., 2018, 2019, 2021). Coherent quantum phase slip can be leveraged through overdamped QPSJs to create individual quantized current pulses, which are analogs to neuron spiking events. When compared to Josephson junction based neuromorphic hardware, simulations of QPSJ neuromorphic circuits have been demonstrated to consume less power and require smaller chip area, all while maintaining a similar operation speed (Cheng et al., 2018). To begin, we briefly review the simulation model and previously reported neuromorphic circuits. We present results from SPICE simulations of multiple new QPSJ-based neuromorphic circuit elements and demonstrate their utility through exploration of a long term depression (LTD) circuit and a long term potentiation (LTP) circuit for use in simplified STDP learning. STDP learning is a form of asynchronous Hebbian learning that uses temporal correlations between the spikes of presynaptic and postsynaptic neurons and is believed to underlie learning and information storage in biological brains (Bi and Poo, 2001). Previously described hardware capable of STDP learning include memristor based approaches (Serrano-Gotarredona et al., 2013) and hybrid superconductive-optoelectronic circuits based on Josephson junctions combined with single photon detectors (Shainline et al., 2019). Though not shown here, neuromorphic circuit elements exhibiting STDP behavior can be systematically connected together to construct a large neural network that is capable of supervised learning with programmable weights using pulsed “write” signals to each synapse or unsupervised learning with long term potentiation and depression circuits. Results from our recent explorations of new versions of these circuits based on QPSJ and QPSJ + JJ are presented in this paper. We also present an alternative approach to construct neural networks by coupling QPSJ-based circuit elements together such that the weights of multiple synapses can be collectively programmed using only a few adjustable parameters. While individual weights cannot be deterministically programmed in such networks, we show that the collective network configuration can be programmed, while the network exhibits STDP learning behavior with respect to the input spiking signals. Therefore, we establish that QPSJ-based neural network elements have the potential to achieve completely supervised and semi-supervised learning, with possibility for unsupervised learning in hardware, which we expect to lead to more capable and lower energy per operation neuromorphic and artificial intelligence systems.

## 2. QPSJ-Based Neuromorphic Circuit Elements

In this section, we briefly re-introduce circuit configurations and principles of operation of a single QPSJ and QPSJ-based neuromorphic circuits to familiarize the readers with QPSJ-based neuromorphic circuits, including neuron, synapse and fan-out circuits (Cheng et al., 2018, 2021). The simulations were carried out in WRspice, using a QPSJ SPICE model introduced in Goteti and Hamilton (2015), along with Python programs to automate a large number of simulations. SPICE is an open-source analog electronic circuit simulator (Nagel, 1975), that performs time-dependent equivalent circuit nodal analysis to determine the resultant electrical behavior. It is worth noting that SPICE is useful for simulating a wide range of dynamic systems (Hewlett and Wilamowski, 2011), including neuromorphic systems.

### 2.1. QPSJ SPICE Model

The equivalent electronic circuit model of a QPSJ is defined by an intrinsic QPSJ in series with a resistor *R* and an inductor *L* (Mooij et al., 2015). The equations that govern QPSJ behavior and are the basis of our SPICE model are:


(1) $$\begin{array}{r} V=V_{\text{c}}\sin\left(q\right)+L\frac{dI}{dt}+RI, \end{array}$$

(2) $$\begin{array}{r} I=\frac{2e}{2\pi }\frac{dq}{dt}, \end{array}$$

where *q* is the charge equivalent in the QPSJ normalized to the charge of a Cooper pair (2*e*). The critical voltage *V*_c_ is defined by:


(3) $$\begin{array}{r} V_{\text{c}}=\frac{2\pi E_{\text{s}}}{2e}, \end{array}$$

where *E*_s_ is the phase-slip energy. The junction exhibits a Coulomb blockade when the applied voltage is below its critical voltage, and becomes resistive when the voltage is above its critical voltage (Hriscu and Nazarov, 2011). The critical voltage is a device parameter, analogs to the critical current of a JJ, which can be tuned through device material, design (i.e., geometrical parameters), and fabrication processes. A single QPSJ can be treated as a series RLC oscillator under appropriate operating conditions. When the oscillator is over-damped, a quantized current pulse (spike) can be generated and propagated, which can be used to emulate neuron spiking behavior. A QPSJ-based neuromorphic system generates, processes and transmits narrow, high-frequency spike-shaped current signals to emulate the dynamics of a biological neural network and perform computational functions based on input and output definitions.

### 2.2. Integrate-and-Fire Neuron

**Figure 1:**
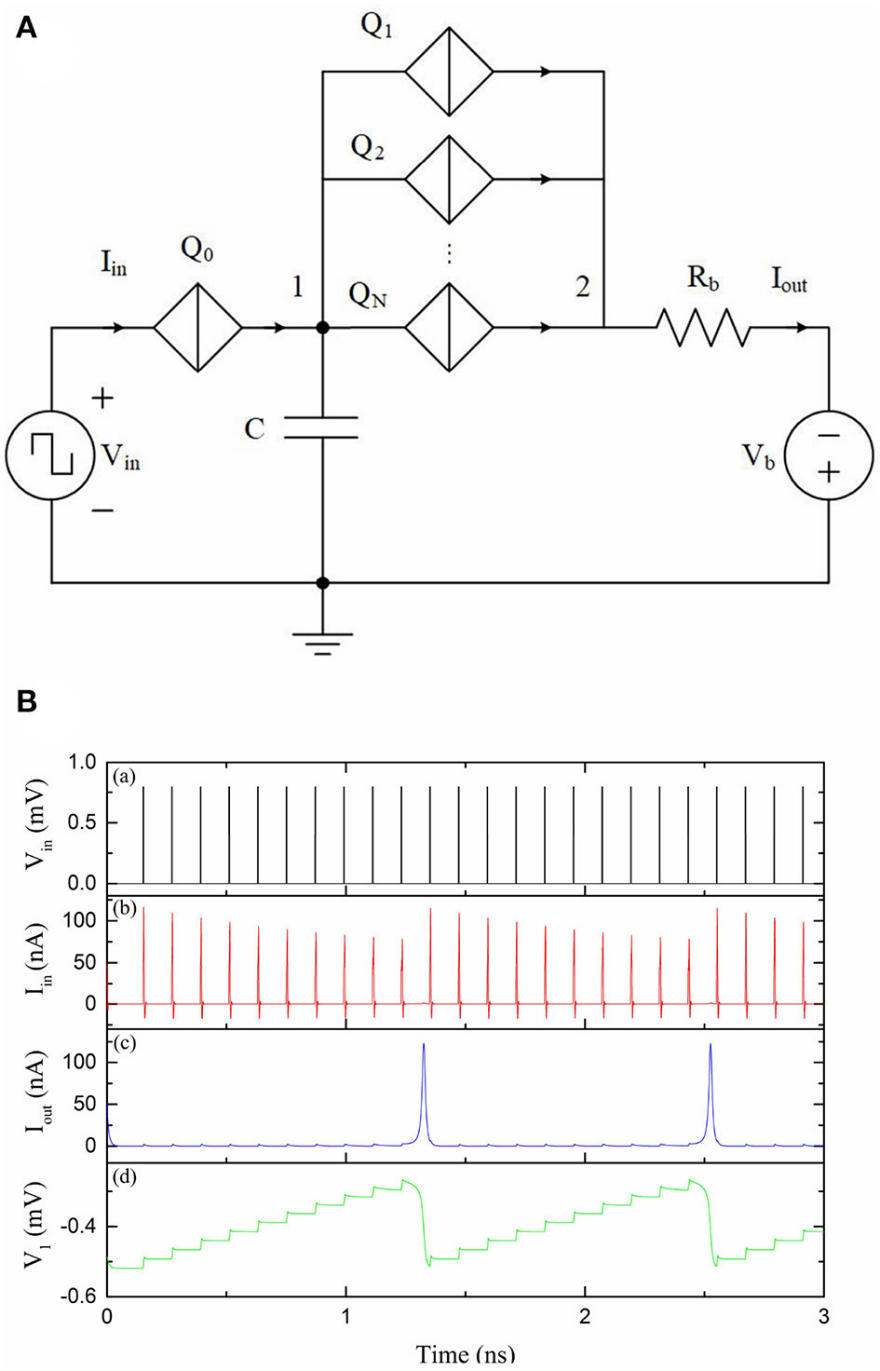
A QPSJ-based IFN circuit that has a firing threshold of *N* (Cheng et al., 2018). *V*_in_ was 0.8 mV and *V*_b_ was 1 mV. The *V*_c_ values used for *Q*_1_–*Q*_*N*_ were 0.7 mV. *R*_*b*_ was 9 kΩ. **(A)** Circuit schematic. **(B)** Simulation results of an IFN circuit with a threshold of 10. (a) Input voltage. (b) Input current. (c) Output current. (d) Voltage at node 1.

Integrate-and-fire neurons (IFNs) perform their neuron function by integrating a signal up to a threshold, after which an output signal (pulse) is generated (fired). A QPSJ-based IFN, as shown in Figure 1A, integrates electrons (through QPSJ *Q*_0_) from input signals, for example from other neurons or control circuitry, onto a membrane capacitor *C* and fires a spike signal if the total number of electrons reach the threshold (Cheng et al., 2018). The threshold is defined in hardware by *N*, the number of parallel QPSJs (*Q*_1_ to *Q*_*N*_) and the capacitance of capacitor *C*, as shown in Figure 1A. The simulation results of this IFN circuit are shown in Figure 1B. The number of parallel QPSJs is 10 (i.e., the threshold is 10) during this simulation. The input voltage pulse from *V*_in_ can switch *Q*_0_ and generate a current pulse that contains a charge of 2*e*. The voltage at capacitor *C* keeps increasing as a result of quantized charge accumulation. Once the voltage applied on the parallel QPSJs reaches the critical voltage, the capacitor discharges a charge of 20*e*. Each 2*e* charge pulse is transmitted through a parallel QPSJ. The ten parallel current pulses are summed at node 2, which results in a current pulse that contains a charge of 20*e*. The circuit operation is similar to a digital IFN circuit that has a pre-defined threshold of *N*. In this example, the time constant associated with discharging of capacitor *C* through normal resistances of the parallel QPSJs is designed to be larger than the switching speed of the QPSJs therefore allowing simultaneous switching of 10 parallel junctions. We note that this parallel combination of nominally identical QPSJs is sensitive to device-to-device variation. While in simulation we can use identical devices, in real hardware, the circuits will have a range of tolerance associated with device-to-device variation. The device-to-device tolerance of these parallel QPSJs was found to be ~1%, according to the simulation results discussed in Cheng et al. (2021). This is an important aspect for advancing this technology and will require close attention in future device fabrication and circuit design efforts. Next, we will briefly introduce a multi-weight synaptic circuit in the following subsection to provide a weighted connection between neuron circuits.

### 2.3. Multi-Weight Synapse

**Figure 2:**
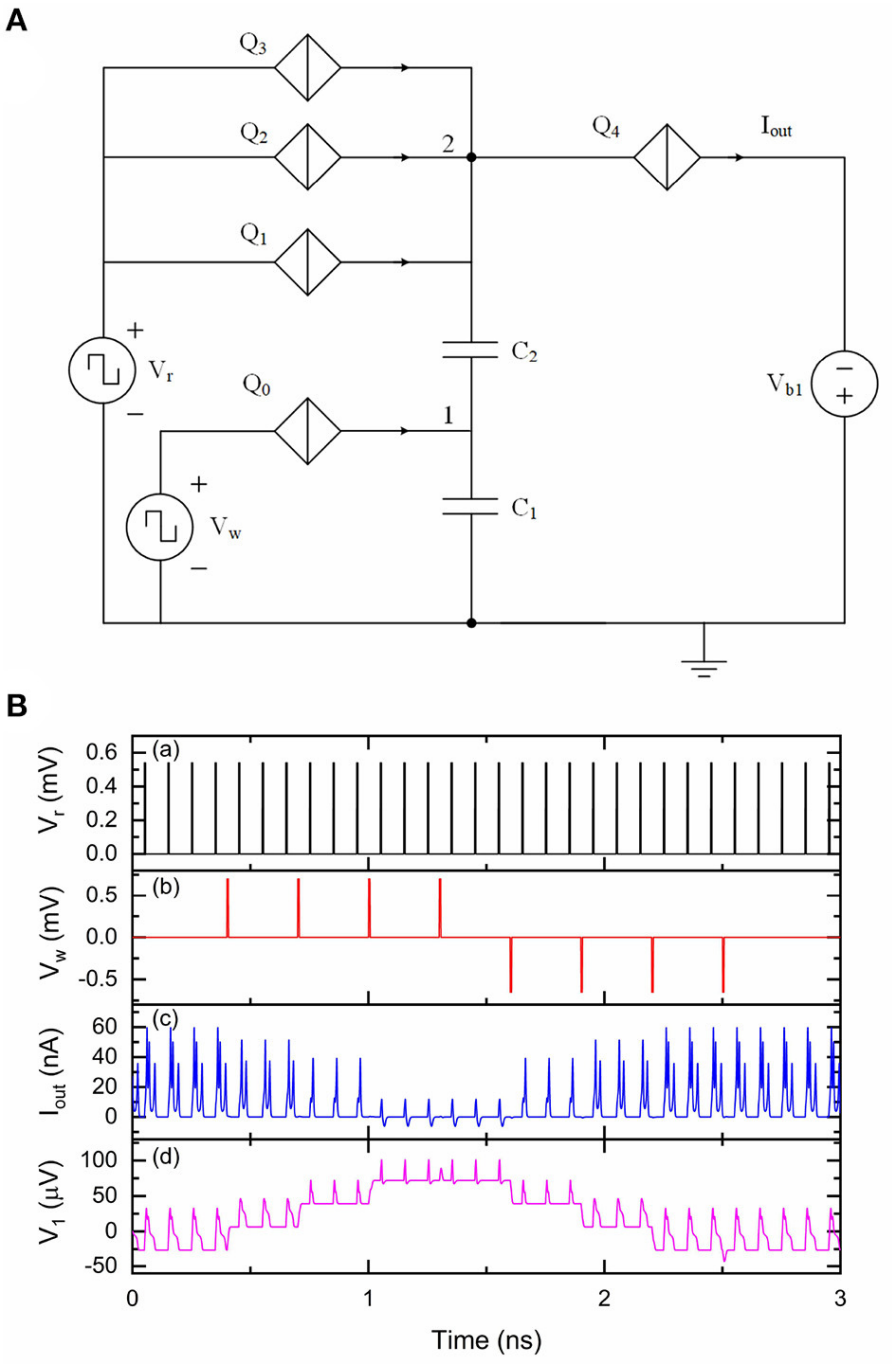
A multi-weight QPSJ-based synaptic circuit that has two inputs *V*_r_ and *V*_w_ and one output *I*_out_. The weight can be modified by applying positive or negative pulses at *V*_w_, and can be read-out by applying positive pulses at *V*_r_. *V*_r_ was 0.54 mV and *V*_w_ was 0.7 mV. The *V*_c_ values used for *Q*_0_–*Q*_4_ were 0.3, 0.5, 0.52, 0.54, and 0.31 mV, respectively. *C*_1_ was 9.2 fF and *C*_2_ was 1.2 fF. *V*_b1_ was 0.5 mV. **(A)** Circuit schematic. **(B)** Simulation results of the synaptic circuit. (a) Read signal *V*_r_. (b) Write signal *V*_w_. (c) Output current *I*_out_. (d) Voltage at node 1.

A synapse is connected between two neurons to transmit weighted spiking signals. We previously designed and presented a QPSJ-based multi-weight synaptic circuit to transmit weighted current pulses between neuron circuits, which is briefly reviewed here (Cheng et al., 2021). The circuit shown in Figure 2A is a multi-weight synaptic circuit that can generate different numbers of sequential current pulses, which correspond to a weight of 0, 1, 2 or 3. Here, the weight is defined as the number of pairs of electrons at the output for each input pulse. In general, *N* sequential current pulses contain *N* pairs of electrons, although the shapes of these pulses may not look significantly different. Parallel QPSJs *Q*_1_, *Q*_2_ and *Q*_3_ have different critical voltages in order to function correctly. The critical voltages of *Q*_1_, *Q*_2_ and *Q*_3_ are *V*_C1_, *V*_C2_ and *V*_C3_, respectively, while *V*_C1_<*V*_C2_<*V*_C3_. Ideally, the critical voltage difference between *Q*_1_ and *Q*_2_ or *Q*_2_ and *Q*_3_ should be the same as the voltage change on node 1 after receiving an input voltage pulse from *V*_w_, which is ~2*e*/*C*_1_. In simulation, the tolerance of these parallel QPSJs was found to be less than ~1% (Cheng et al., 2021). The weight can be increased by applying negative pulses at *V*_w_ or decreased by applying positive pulses at *V*_w_. Applying (positive) pulses at *V*_r_ can read but not destroy the neuron memory state. Different numbers of sequential current pulses will be generated at *I*_out_ upon the arrival of one short voltage pulse at *V*_r_, depending on the number of electrons stored at capacitor *C*_1_. The simulation results of this circuit are shown in Figure 2B. The initial weight of the synaptic circuit is set to 3. A voltage pulse from *V*_*r*_ can switch all three parallel QPSJs *Q*_1_, *Q*_2_, and *Q*_3_, resulting in three sequential current pulses at *I*_out_. Applying a positive voltage pulse at *V*_w_ can add two electrons onto capacitor *C*_1_ and the voltages at node 1 and node 2 increase accordingly. In this case, the upcoming voltage pulse from *V*_*r*_ can only switch two out of three parallel QPSJs, which causes two sequential current pulses at *I*_out_. Once the synaptic weight reaches 0, it will not decrease any more. Similarly, applying a negative voltage pulse at *V*_w_ can take two electrons from capacitor *C*_1_ and the voltages at node 1 and node 2 decrease accordingly. Therefore, the synaptic weight is increased by 1. This can be repeated up to reaching the maximum weight. Different weights result in different numbers of sequential current pulses at *I*_out_ during each read operation. The weight modulation scheme in this circuit allows us to design learning circuits that can generate appropriate positive and negative pulses based on specific learning rules to control the synaptic weight.

### 2.4. Fan-Out Circuit

**Figure 3:**
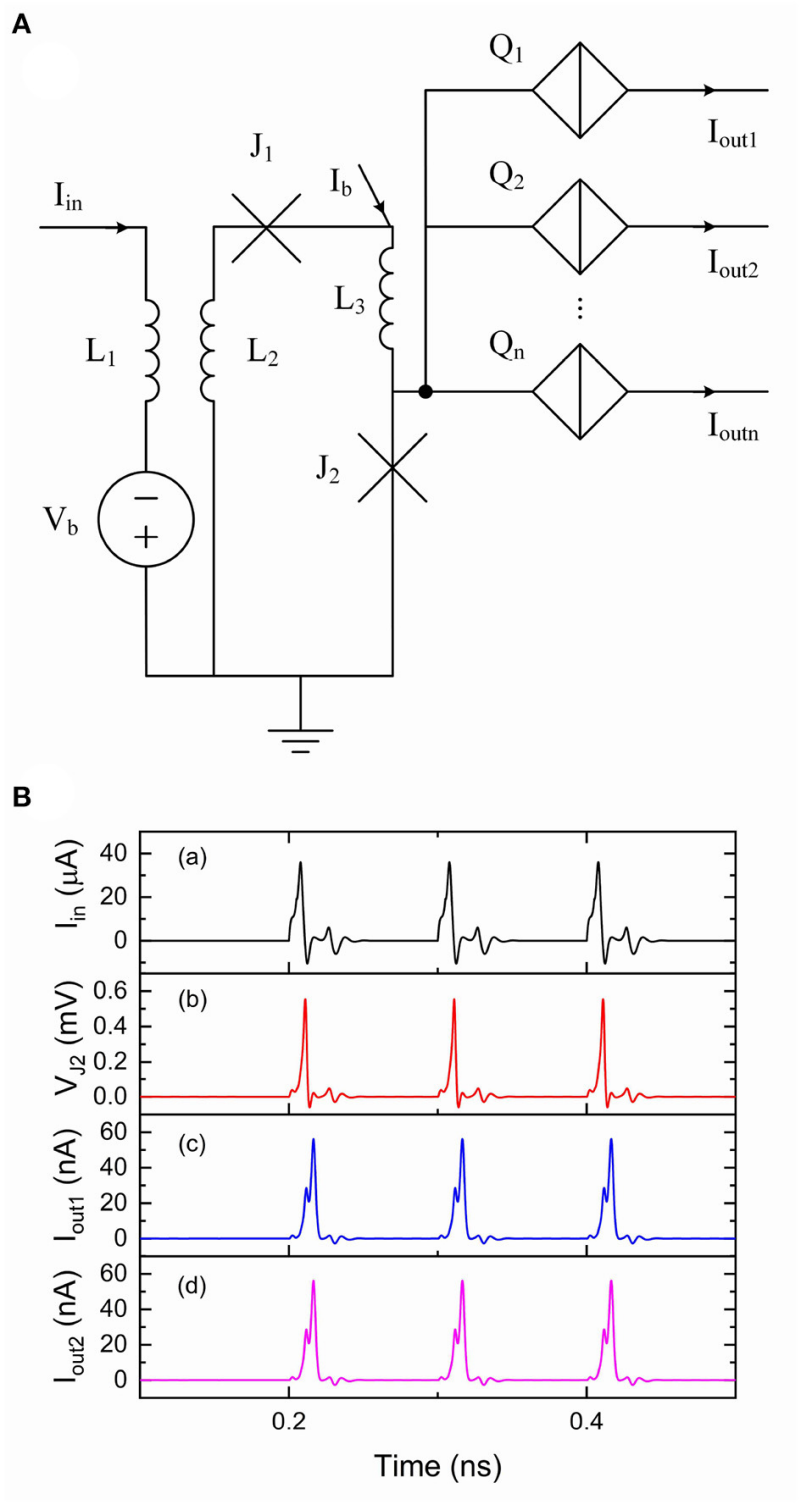
A fan-out circuit is comprised of flux-charge and charge-flux circuits (Cheng et al., 2021). The *V*_c_ values used for *Q*_1_ to *Q*_10_ were 0.5 mV. The critical current *I*_c_ value used for *J*_1_ was 40 μ*A* and the *I*_c_ value used for *J*_2_ was 50 μ*A*. The inductance values used for *L*_1_ and *L*_2_ were 0.1 nH with a coupling coefficient of 0.9. The inductance value used for *L*_3_ was 0.01 nH. Bias current *I*_b_ was 70 μ*A* and bias voltage *V*_b_ was 0.5 mV. Input current *I*_in_ was from the output of a QPSJ-based IFN circuit that has a threshold of 500. **(A)** Circuit schematic. **(B)** Simulation results of the fan-out circuit. (a) Input current. (b) Voltage at *J*_2_. (c) Output current 1. (d) Output current 2.

In biological neural systems, neurons are typically connected to thousands of other neurons (von Bartheld et al., 2016). A fan-out circuit allows one neuron to be able to connect to multiple other neurons. We previously designed and presented a fan-out circuit to split current pulses from an IFN circuit to provide a means to connect to other IFN circuits, which is briefly reviewed here (Cheng et al., 2021). Charge-flux converters (Goteti and Hamilton, 2019) were used to convert quantized current pulses to single flux quantum (SFQ) pulses that can in turn switch multiple QPSJs, as shown in Figure 3A. The current pulse *I*_in_ from an IFN circuit flows into an inductor *L*_1_. The current pulse is then coupled to a mutual inductor *L*_2_ and injected to Josephson junctions *J*_1_ and *J*_2_. Since *J*_2_ is biased to a value near its critical current by bias current *I*_b_, the additional current pulse from *L*_2_ can switch *J*_2_ and generate a SFQ pulse, which can in turn switch multiple parallel QPSJs. Once *J*_2_ is switched and in the resistive state, *J*_1_ can be switched by *I*_b_ and the system recovers to its initial state. This circuit can be designed to provide a large fan-out. As an example, the simulation results of a ten fan-out circuit are shown in Figure 3B. We use an IFN circuit that has a threshold of 500 to generate current pulses flowing into *I*_in_. The induced current pulses from mutual inductors are injected to *J*_2_, resulting in SFQ pulses across *J*_2_. Since all the parallel QPSJs (*Q*_1_ to *Q*_10_) are switched at the same time, we can see identical output current pulses from *I*_out1_ and *I*_out2_ in Figure 3Bc,d, which are synchronized to the input current pulses. The simulation results have demonstrated the fan-out function of this circuit. This circuit does not appear to have a limit for the maximum fan-out in simulation, but can be limited by the practical circuits due to fabrication challenges (Cheng et al., 2021).

## 3. QPSJ-Based Learning Circuits

The human brain can be viewed as an energy-efficient learning machine, solving demanding computational tasks while consuming a small amount of energy. One feature of the human brain that enables it to adapt to the surrounding environment and to solve complex problems is synaptic plasticity (Haykin, 2010). In order to mimic this synaptic plasticity in neuromorphic computing we desire to have the ability to adjust synaptic weights through learning processes. While there are multiple learning strategies in neuromorphic computing, we focused on the STDP learning approach in this work to provide potential learning functions for QPSJ-based superconductive neuromorphic systems. Early neuroscience experiments on synaptic plasticity suggested that the relative timing of presynaptic and postsynaptic action potentials, on a timescale of milliseconds, had significant effects on the plasticity (Levy and Steward, 1983). This is well known as spike timing dependent plasticity (STDP), which was observed in cortical neurons (Cooke and Bliss, 2006). In neuromorphic hardware systems, STDP-type learning rules are widely used as an unsupervised learning method (Linares-Barranco et al., 2011; Lee et al., 2018; Srinivasan et al., 2018). In this paper, we introduce a method of realizing a simplified STDP rule using QPSJ-based circuits. The weight change is either +1 or −1 during each learning event. The learning circuit is comprised of a long term depression circuit and a long term potentiation circuit, which are combined together to realize a simplified STDP rule circuit. Each of these circuits are described in more detail in the following sections.

### 3.1. A Long Term Depression Circuit

**Figure 4:**
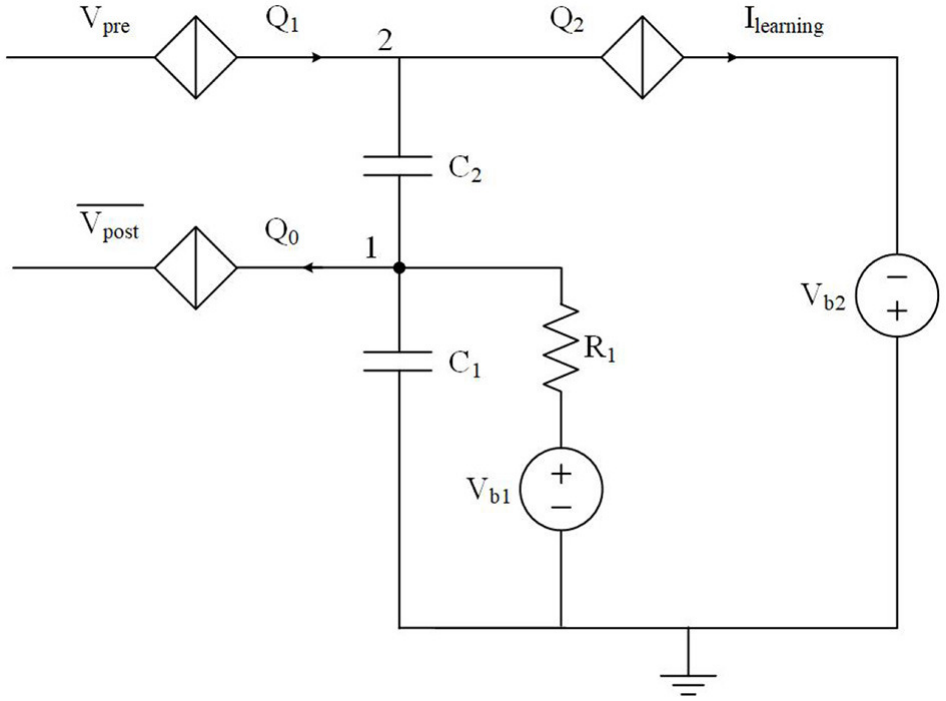
An LTD circuit that generates depression pulses to a synapse. A pulse will be generated at *I*_learning_ when the timing difference Δ*t* = *t*_post_ #x02212; *t*_pre_ is within a short learning window.

**Figure 5:**
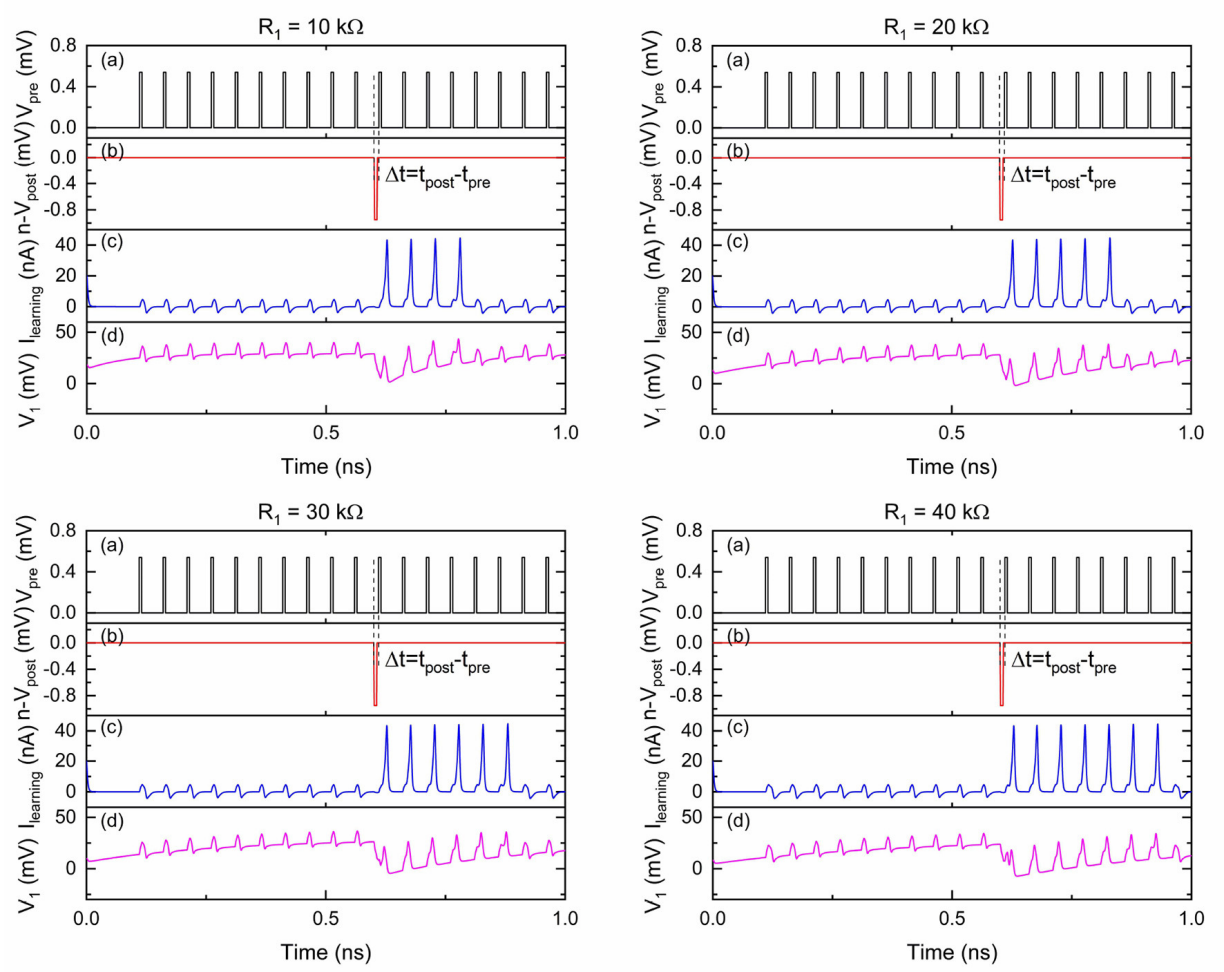
Simulation results of the circuit shown in Figure 4 with different *R*_1_ values. *V*_pre_ was 0.54 mV and $\bar{V_{\text{post}}}$ was 0.95 mV. The critical voltage values used for *Q*_0_–*Q*_2_ were 0.75, 0.56, and 0.31 mV, respectively. *C*_1_ was 9.2 fF and *C*_2_ was 1.2 fF. *V*_b1_ was 0.03 mV and *V*_b2_ was 0.5 mV. *R*_1_ was 10/20/30/40 kΩ. (a) Input signal *V*_pre_. (b) Input signal $\bar{V_{\text{post}}}$. (c) Output signal *I*_learning_. (d) Voltage at node 1.

In a biological neural system, long term depression (LTD) occurs when a postsynaptic spike leads a presynaptic spike by ~20–100 ms (Ito and Kano, 1982; Markram et al., 1997). The synaptic weight between these two neurons is thus depressed as they are considered to be uncorrelated. The LTD circuit shown in Figure 4 can generate positive pulses used to depress the synaptic weight if the timing difference Δ*t* = *t*_post_ − *t*_pre_ is within a short learning window. This circuit operates at a much faster speed than its biological counterpart, tens of GHz vs. kHz, therefore LTD is designed to be effective within a shorter (ps scale) learning window. In Figure 4, the initial voltage at node 1 is set by bias voltage *V*_b1_ when there are no inputs at $\bar{V_{\text{post}}}$. In the circuit design, we choose an appropriate critical voltage value for *Q*_1_ such that *Q*_1_ cannot be switched by *V*_pre_ should a voltage pulse from *V*_pre_ arrive first. Therefore, no current pulses are generated at *I*_learning_. On the other hand, if a negative voltage pulse from $\bar{V_{\text{post}}}$ arrives first, *Q*_0_ is switched and a pair of electrons are taken from capacitor *C*_1_. The voltages at node 1 and node 2 drop by 2*e*/*C*_1_, where *C*_1_ is the capacitance of capacitor *C*_1_. The slight voltage change at node 2 allows the upcoming pulse from *V*_pre_ to switch *Q*_1_ and in turn switch *Q*_2_, resulting in a positive current pulse at *I*_learning_. The voltages at nodes 1 and 2 will recover to their initial states since *C*_1_, *R*_1_, and *V*_b1_ behave like a series RC circuit with a corresponding voltage decay time. As a result, there will be pulses at *I*_learning_ only if signals at *V*_pre_ and $\bar{V_{\text{post}}}$ are close enough in time. The width of the learning window is determined by the resistance value of *R*_1_. The simulation results in Figure 5 illustrate how the learning window changes as *R*_1_ changes.

In Figure 5, the voltage at node 1 drops upon arrival of a negative pulse into $\bar{V_{\text{post}}}$. A current pulse at *I*_learning_ is followed by each upcoming pulse from *V*_pre_ before the voltage at node 1 gradually increases to a stable point. The effective time window over which the circuit responds as intended is viewed as the learning window for this LTD function. In this LTD circuit design, the width of the learning window increases as *R*_1_ increases. This can be explained by the different voltage level recovering speeds due to different RC time constants.

**Figure 6:**
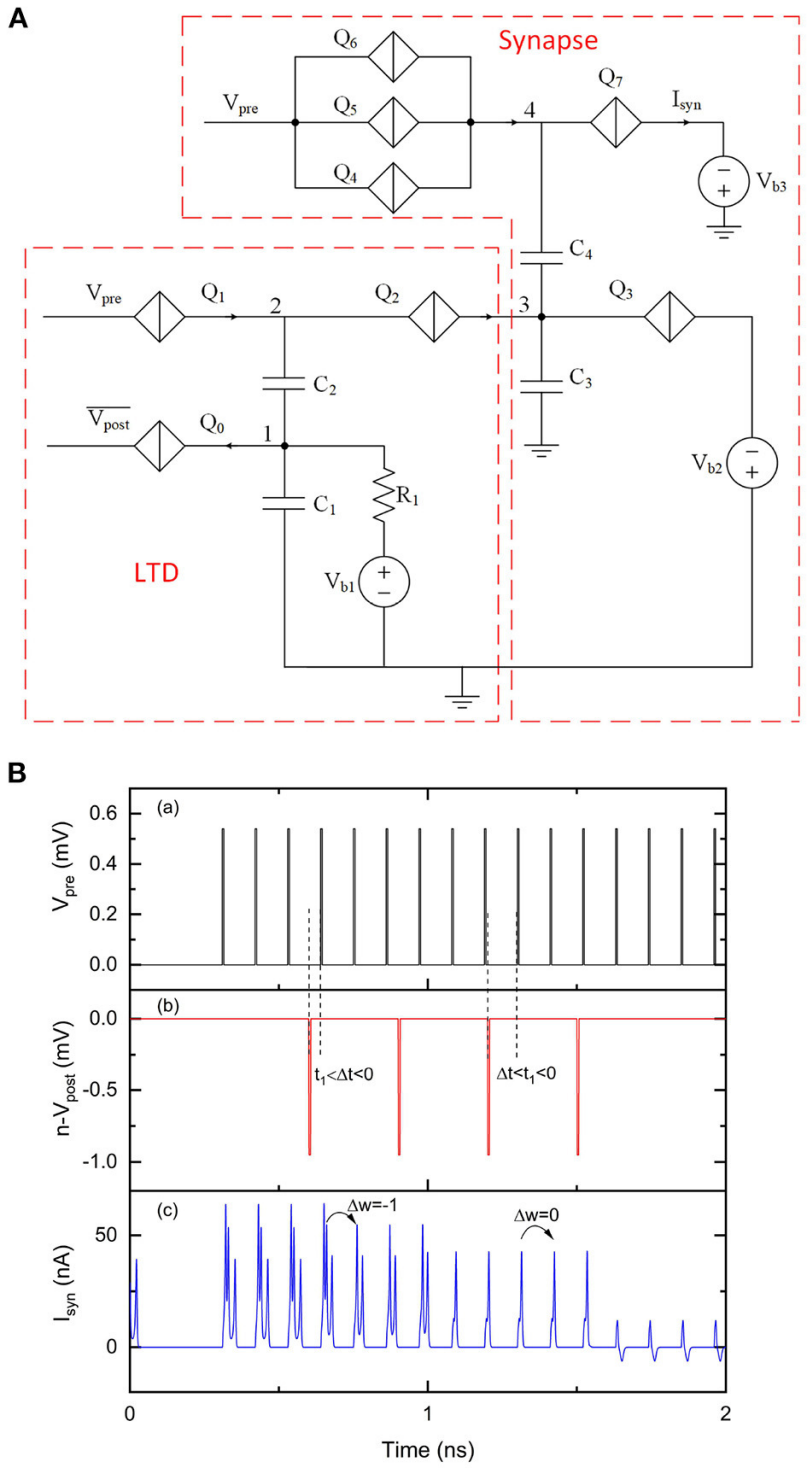
An LTD circuit with a multi-weight synaptic circuit. The number of sequential current pulses at *I*_syn_ can be reduced when the timings of pulses from *V*_pre_ and $\bar{V_{\text{post}}}$ trigger an LTD learning event. *V*_pre_ was 0.54 mV and $\bar{V_{\text{post}}}$ was 0.95 mV. The critical voltage values used for *Q*_0_–*Q*_7_ were 0.75, 0.55, 0.3, 2, 0.54, 0.52, 0.5, and 0.34 mV, respectively. *C*_1_ and *C*_3_ were 9.2 fF, and *C*_2_ and *C*_4_ were 1.2 fF. *V*_b1_, *V*_b2_ and *V*_b3_ were 0.03, 0.77, and 0.53 mV, respectively. *R*_1_ was 10 kΩ. (a) Input signal *V*_pre_. **(A)** Circuit schematic. **(B)** Simulation results. (a) Input signal *V*_pre_. (b) Input signal $\bar{V_{\text{post}}}$. (c) Output signal *I*_syn_.

This LTD circuit works seamlessly with a synaptic circuit as shown in Figure 6A. LTD occurs when the circuit detects *t*_1_ < Δ*t* < 0, where *t*_1_ ≈ 50 ps defines the maximum LTD learning window. Charge (electrons) will be injected onto capacitor *C*_3_, which depresses the synaptic weight. A simulation was performed to show how the synaptic weight changes according to the LTD rule. The results are shown in Figure 6B. The width of LTD learning window was not a concern during this simulation, as the circuit parameters were chosen to demonstrate LTD functions but not for a specific LTD learning window. In this circuit design, the initial weight was set to 3 based on the device parameters used for this simulation. Each presynaptic pulse could result in three sequential current pulses (containing a charge of six electrons) at *I*_syn_. As the first negative voltage pulse from $\bar{V_{\text{post}}}$ is presented, the voltage at node 1 drops due to the switching of *Q*_0_, which takes two electrons from *C*_1_. The voltage at node 2 also drops subsequently, which allows the fourth voltage from *V*_pre_ to switch *Q*_1_ and in turn switch *Q*_2_ to inject two electrons onto *C*_3_. As a result, the synaptic weight is depressed by 1. The weight change is not immediate but can be observed by the upcoming pulse from *V*_pre_, which results in two sequential current pulses (containing a charge of four electrons) at *I*_syn_. We can also see that the timings between the third pulse from $\bar{V_{\text{post}}}$ and the tenth pulse from *V*_pre_ is relatively larger (~100 ps), which does not result in a weight depression. This is because the voltage at node 1 and node 2 recover to their initial states (set by bias voltages) before the tenth pulse from *V*_pre_ arrives. These simulation results demonstrate that the LTD circuit can realize a weight depression function with respect to the relative timing information between presynaptic and postsynaptic pulses.

### 3.2. A Long Term Potentiation Circuit

**Figure 7:**
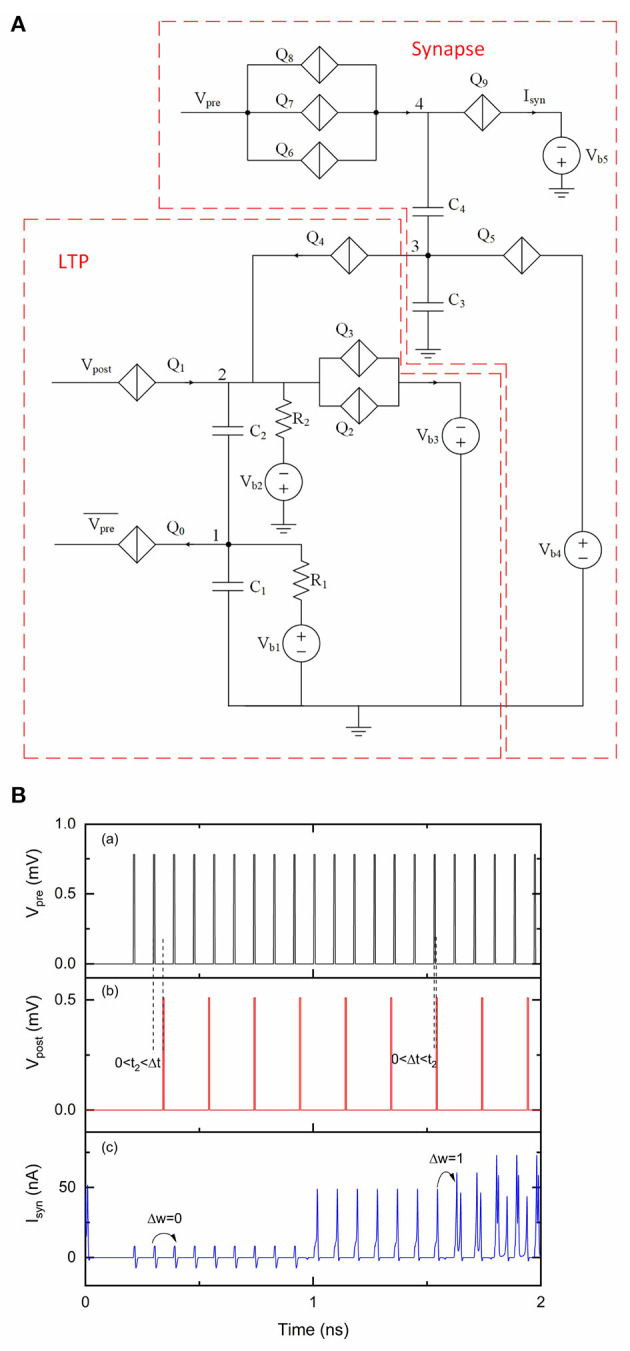
An LTP circuit with a multi-weight synaptic circuit. The number of sequential current pulses at *I*_syn_ can be increased when the timings of pulses from *V*_pre_ and *V*_post_ trigger an LTP learning event. *V*_pre_ was 0.78 mV and $\bar{V_{\text{pre}}}$ was 0.54 mV. *V*_post_ was 0.51 mV. The critical voltage values used for *Q*_0_–*Q*_9_ were 0.4, 0.5, 1, 1, 0.58, 2, 1.04, 1.02, 1, and 0.28 mV, respectively. *C*_1_, *C*_2_, *C*_3_, and *C*_4_ were 9 fF, 1 fF, 9.2 fF and 2 fF, respectively. *V*_b1_, *V*_b2_, *V*_b3_, *V*_b4_ and *V*_b5_ were 0.05, 0.2, 1.1, 1.01, and 0.6 mV, respectively. *R*_1_ and *R*_2_ were 10 kΩ. **(A)** Circuit schematic. **(B)** Simulation results. (a) Input signal *V*_pre_. (b) Input signal *V*_post_. (c) Output signal *I*_syn_.

In a biological neural system, long term potentiation (LTP) occurs when a presynaptic spike leads a postsynaptic spike by up to 20 ms (Bliss and Lømo, 1973; Markram et al., 1997). The synaptic weight between these two neurons is thus potentiated as they are considered as correlated. In a synaptic circuit shown in Figure 2, the weight can be potentiated by applying negative pulses at *V*_w_. Here we propose an LTP circuit that can generate negative current pulses to potentiate the synaptic weight according to the relative timing information between a presynaptic neuron and a postsynaptic neuron. The circuit shown in Figure 7A is an LTP circuit with a multi-weight synaptic circuit. Similar to the LTD circuit shown in Figure 4, the LTP circuit has two inputs $\bar{V_{\text{pre}}}$ and *V*_post_. *Q*_2_ and *Q*_3_ are identical and biased by voltage *V*_b3_. The initial voltage at node 1 is set by bias voltage *V*_b1_. Voltage at node 2 (*V*_2_) is set by bias voltage *V*_b2_ so that the voltage across *Q*_2_ and *Q*_3_ is near their critical voltages. When there are no inputs at $\bar{V_{\text{pre}}}$, *V*_post_ cannot switch *Q*_1_. A negative voltage pulse from $\bar{V_{\text{pre}}}$ can switch *Q*_0_, taking two electrons from capacitor *C*_1_. The voltage drop at node 1 results in a voltage drop at node 2 as well. The slight voltage change at node 2 allows the upcoming voltage pulse from *V*_post_ to switch *Q*_1_ and in turn switch *Q*_2_ and *Q*_3_, resulting in a current pulse that contains a charge of 4*e*. Since there are only two electrons coming from *V*_post_, the voltage drop at node 2 allows *Q*_4_ to be switched and allows *C*_3_ to provide another pair of electrons. This circuit behaves like an “inverter” circuit that can convert positive voltage (or current from an upstream neuron) pulses to negative current pulses. By choosing appropriate biasing conditions and critical voltage value of *Q*_4_, we only allow *Q*_4_ to be switched for a maximum of three times, which represents a maximum weight change of 3. Each time *Q*_4_ is switched, a pair of electrons flow from *C*_3_ to *C*_2_ and voltage at node 3 drops by 2*e*/*C*_3_, which makes the synaptic weight increase by 1.

In Figure 7B, we assume LTP is effective when 0 < Δ*t* < *t*_2_, where *t*_2_ ≈ 34 ps is primarily determined by the resistance of *R*_1_ in Figure 7A. The width of LTP learning window was not a concern during this simulation, as the circuit parameters were chosen to demonstrate LTP functions but not for a specific LTP learning window. The initial weight of the synapse was set to 0. Different periodic pulses were applied at *V*_pre_ and *V*_post_ in the simulation to demonstrate LTP learning. For example, the sixteenth pulse from *V*_pre_ is slightly ahead of the seventh pulse from *V*_post_, which triggers LTP for the multi-weight synapse. As a result, the weight changes from 1 to 2. The upcoming pulse from *V*_pre_ can trigger two sequential current pulses at *I*_syn_. However, the second pulse from *V*_pre_ has a relatively large time interval (~100 ps) with the first pulse from *V*_post_, which does not trigger a weight change.

**Figure 8:**
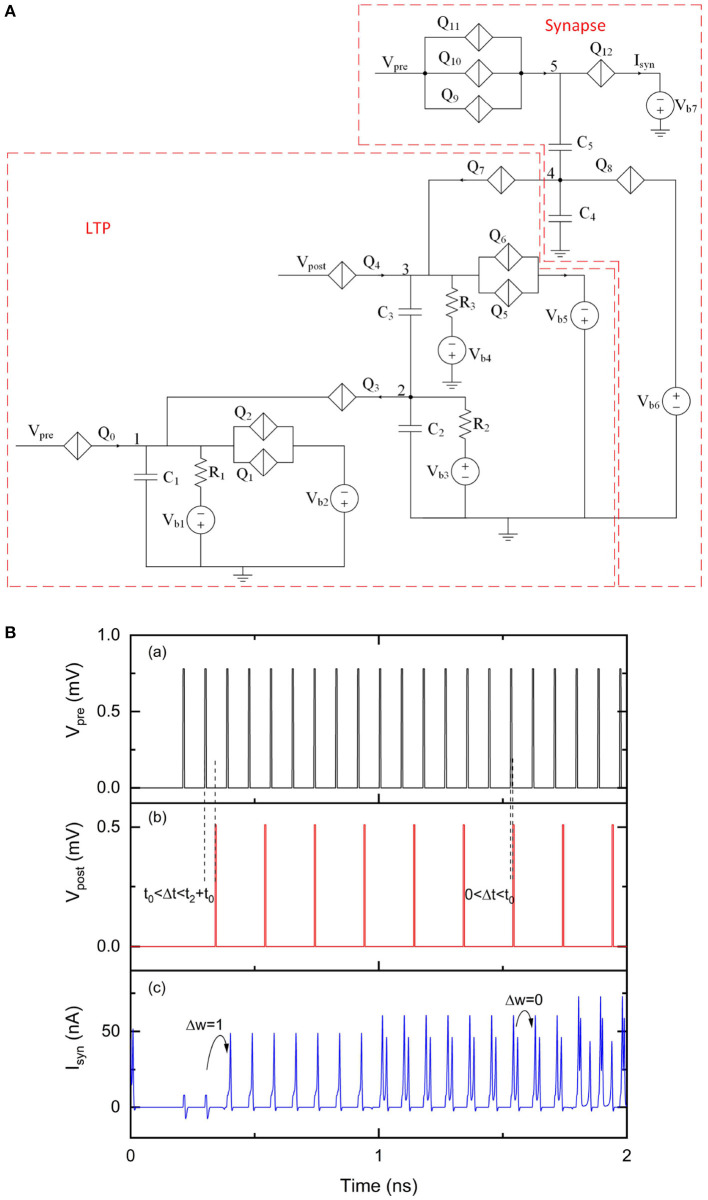
A modified LTP circuit with a multi-weight synaptic circuit. The number of sequential current pulses at *I*_syn_ can be increased when the timings of pulses from *V*_pre_ and *V*_post_ trigger an LTP learning event. *V*_pre_ was 0.78 mV and *V*_post_ was 0.51 mV. The critical voltage values used for *Q*_0_–*Q*_12_ were 0.8, 0.95, 0.95, 0.36, 0.5, 1, 1, 0.58, 2, 1.04, 1.02, 1, and 0.28 mV, respectively. *C*_1_, *C*_2_, *C*_3_, *C*_4_, and *C*_5_ were 1, 9, 1, 9.2, and 2, respectively. *V*_b1_, *V*_b2_, *V*_b3_, *V*_b4_, *V*_b5_, *V*_b6_, and *V*_b7_ were 0.2, 1.1, 0.05, 0.2, 1.1, 1.01, and 0.6 mV, respectively. *R*_1_, *R*_2_, and *R*_3_ were 10 kΩ. **(A)** Circuit schematic. **(B)** Simulation results. (a) Input signal *V*_pre_. (b) Input signal *V*_post_. (c) Output signal *I*_syn_.

We replaced negative input voltage pulses from $\bar{V_{\text{pre}}}$ with positive input voltage pulses from *V*_pre_ in the circuit shown in Figure 8A. This circuit contains another “inverter” circuit to convert positive voltage pulses from *V*_pre_ to negative current pulses. As we explained earlier, the “inverter” circuit can take electrons from capacitor *C*_2_ to temporally reduce voltage at node 2. Like many other technologies, signals transmission and processing in QPSJ-based circuits exhibit delays. The extra “inverter” circuit in Figure 8A also adds extra delay. The learning window shifts by *t*_0_≃ 10 ps and becomes ~*t*_0_ < Δ*t* < *t*_2_ + *t*_0_, as shown in Figure 8B. Although the input signals are identical during the simulations, the output results of *I*_syn_ in Figure 8B are different from results in Figure 7B. We observed that LTP occurs in Figure 8B where Δ*t* is relatively large (*t*_2_ < Δ*t*<*t*_2_+*t*_0_) but does not occur where Δ*t* is very small (0 < Δ*t* < *t*_0_). Proper choice of resistance values and potentially adding a delay circuit (e.g., using a QPSJ transmission line circuit) for some of the input signals can adjust the LTP learning window to desired values.

### 3.3. A Spike Timing Dependent Plasticity Circuit

**Figure 9:**
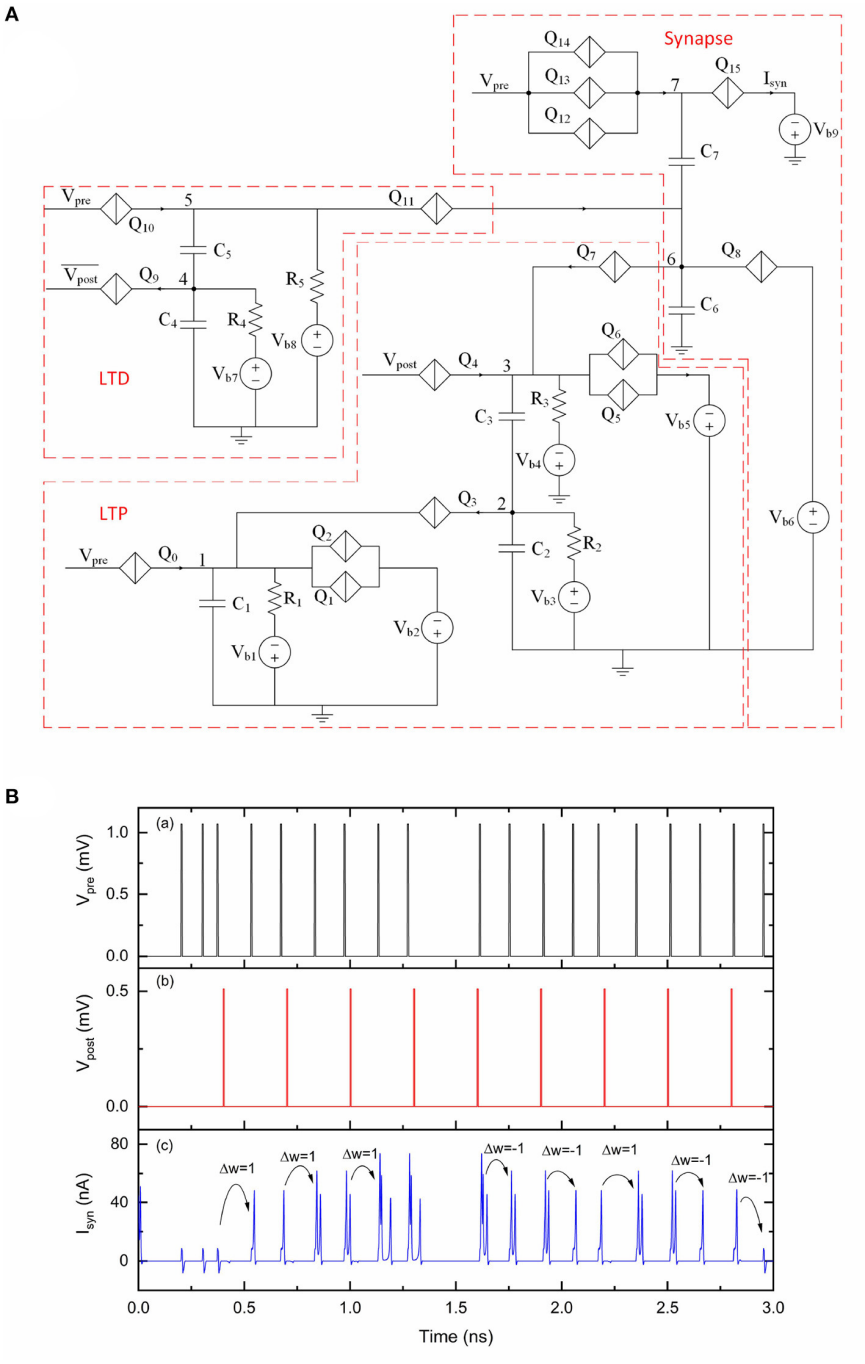
An STDP circuit with a multi-weight synaptic circuit. The number of sequential current pulses at *I*_syn_ can be updated according to the timings of pulses from *V*_pre_ and *V*_post_. *V*_pre_ was 1.07 mV. *V*_post_ was 0.51 mV and $\bar{V_{\text{post}}}$ was 0.51 mV. The critical voltage values used for *Q*_0_–*Q*_15_ were 0.8, 0.95, 0.95, 0.36, 0.5, 1, 1, 0.46, 2, 0.75, 0.55, 0.3, 1.37, 1.35, 1.33, and 0.28 mV, respectively. *C*_1_, *C*_2_, *C*_3_, *C*_4_, *C*_5_, *C*_6_, and *C*_7_ were 1, 9, 1, 9.2, 1.2, 9.2, and 2 fF, respectively. *V*_b1_, *V*_b2_, *V*_b3_, *V*_b4_, *V*_b5_, *V*_b6_, *V*_b7_, *V*_b8_ and *V*_b9_ were 0.2, 1.1, 0.05, 0.2, 1.1, 0.89, 0.46, 0.3, and 0.6 mV, respectively. *R*_1_, *R*_2_, *R*_3_, *R*_4_, and *R*_5_ were 10, 10, 20, 10, and 20 kΩ, respectively. **(A)** Circuit schematic. **(B)** Simulation results. (a) Input signal *V*_pre_. (b) Input signal *V*_post_. (c) Output signal *I*_syn_.

A simplified STDP rule can be realized by combining the proposed LTD and LTP circuit, as shown in Figure 9A. Charge can be injected onto or taken from capacitor *C*_6_, resulting in a weight depression or potentiation for the multi-weight synapse. The LTD portion has an additional bias voltage *V*_b8_ and a resistor *R*_5_ to provide voltage bias for *Q*_10_ and *Q*_11_, which is different from the original LTD circuit shown in Figure 4. The simulation results of this circuit are shown in Figure 9B. We use a customized spike train applied at *V*_pre_ and a periodic spike train applied at *V*_post_ and $\bar{V_{\text{post}}}$ to demonstrate STDP learning functionality. The initial weight of the synapse is set to 0. At the beginning of this simulation, no current pulses are presented at *I*_syn_ when applying voltage pulses at *V*_pre_. As synaptic weight changes according to the relative timings of presynaptic pulses and postsynaptic pulses, the output current pulses at *I*_syn_ also change over time. Specifically, both presynaptic and postsynaptic voltage pulses are transmitted to the LTD and LTP units. However, using the specific device parameter values during this simulation, the LTD unit only generates depression pulses to the synaptic circuit if −10ps < Δ*t* < −2ps. The LTP unit only generates potentiation pulses to the synaptic circuit if 16ps < Δ*t* <41ps. These results demonstrate the simplified learning rule realized by this STDP circuit.

The simplified learning rule presented in this paper aims to provide a simple learning method to update synaptic weights according to relative timings of presynaptic and postsynaptic pulses, but has interesting differences compared to its biological counterpart. One aspect is that the superconducting circuit processes information for signals with pulse rates in the tens of GHz scale, which is many orders of magnitude faster than a human brain that typically operates at tens of Hz. Another aspect is the effective learning window for a circuit in Figure 9A is −10 to −2 ps for LTD and 16 to 41 ps for LTP using the specific parameters in this simulation. Though this learning window does not have the exact shape of a more realistic STDP, it may still be useful for implementation to solve practical problems. We also note that the learning window can be adjusted by slightly modifying the STDP circuit in Figure 9A, in addition to what we mentioned earlier to fix delay issues noted in this paper. For example, adding QPSJs in parallel with *Q*_9_ and increasing the resistance of *R*_4_ could extend the effective learning window for LTD. We have not yet combined input and output neuron circuits, synaptic circuit, fan-out circuit and STDP circuit to demonstrate a large network application. While voltage biasing in QPSJ-based circuits has advantages, as circuit sizes grow and become more complex, challenges related to biasing and impedance matching will likely become more critical (Cheng et al., 2021). We believe that these challenges, which are also found as challenges in other technologies (e.g., current distribution in large JJ-based circuits), do have engineering solutions and require additional work. We also note that these solutions may exist as trade-offs with circuit operation speed and may impact the overall power or energy efficiency. Circuit modifications and new circuit configurations to realize interconnection circuits for synapse feedback loops may also be needed. These aspects are expected to be the focus of potential improvements in future studies.

## 4. Neural Networks With Coupled Charge-Island Synapses

In the previous sections, we have introduced multi-weight synapse circuits (Figure 2) where the weight can either be programmed using voltage pulses *V*_w_ for supervised learning, or can be coupled to long term potentiation and depression circuits to form of an STDP circuit (Figure 9A) as a route to achieving unsupervised learning. Such synapses can be connected to neurons (Figure 1) to construct neural networks with the ability to program individual synapses. While this approach to neural networks is desirable for several applications, it is also possible to construct simpler neural networks using fewer circuit elements by coupling several individual dissimilar synaptic elements together analogs to neural network architectures presented in Goteti et al. (2021) and Goteti and Dynes (2021). This approach takes advantage of the exponential scaling of memory capacity with size that arises from complex (and possibly random) connectivity between nodes in the network similar to that of biological neural networks (Hopfield and Herz, 1995). While such coupled synapses cannot be programmed individually, the circuit construction comprises a mechanism to simultaneously update the weights of all the synapses in the network using only a small number of bias voltage terminals. Additionally, we show that this approach allows weights to be programmed within a continuous set of values, and therefore shows potential to be robust to variation and noise associated with wider device parameter margins. Therefore, this approach may be useful in certain applications to implement aspects of spike-timing and rate-dependent plasticity, with algorithms implemented through coupling of bias voltages to the output signals. Furthermore, as an example, small randomly connected networks could also be used as multi-weight synapse components in larger specifically organized networks, though this is not explored in this work.

**Figure 10:**
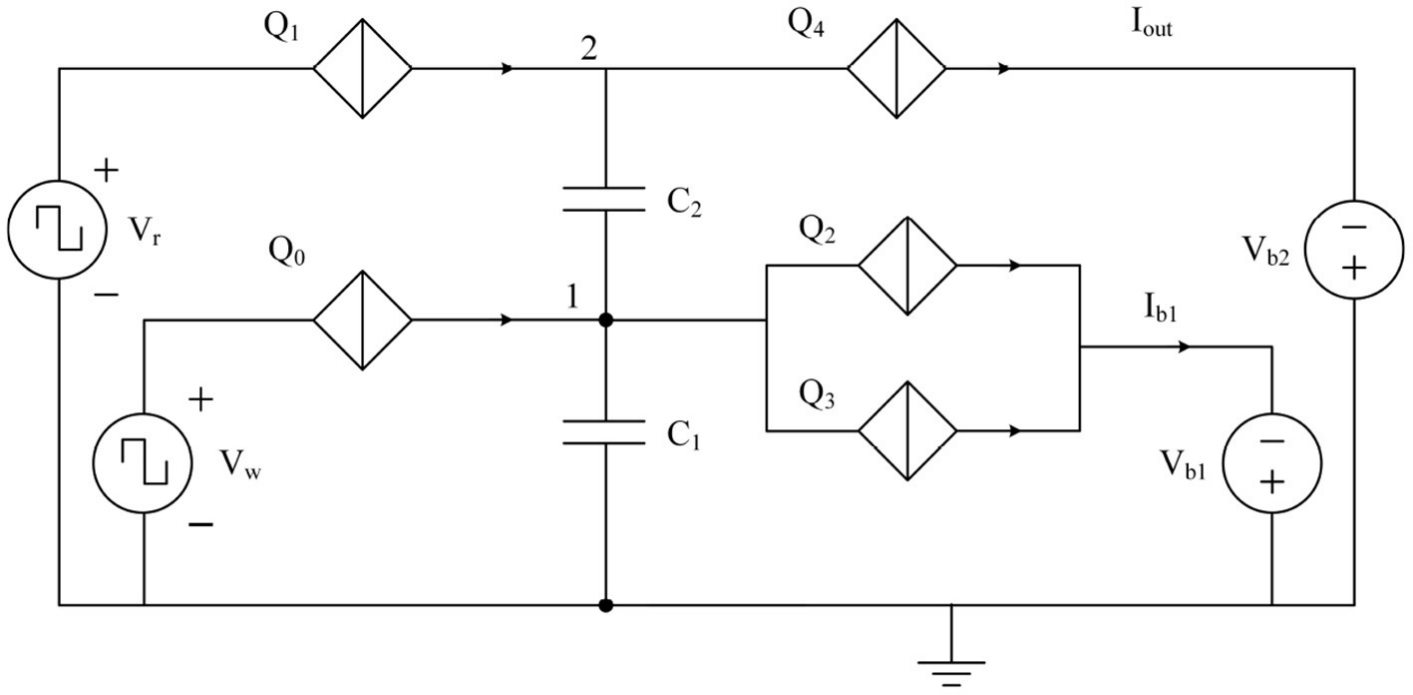
Binary synapse circuit reported in Cheng et al. (2021) reconfigured to operate as a 2 × 2 synapse network.

**Figure 11:**
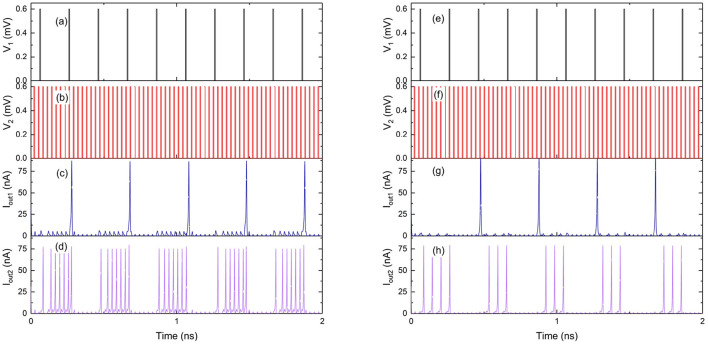
Simulation results of the 2 × 2 synapse network shown in Figure 10. **(a–d)** Capacitors *C*_1_ and *C*_2_ are both chosen to be 3*fF*. **(e–h)** Capacitors *C*_1_ and *C*_2_ are both chosen to be 6*fF*. **(a,e)** Voltage pulse input at *V*_w_ of the binary synapse circuit shown in Figure 10. **(b,f)** Voltage pulse input at *V*_r_. **(c,g)** Current output at *I*_*b*1_. **(d,h)** Current output at *I*_out_.

The approach to a coupled synapse network can be demonstrated using binary synapse circuits previously introduced in Cheng et al. (2021). An equivalent circuit that can operate as a simplified 2 × 2 synapse-network is shown in Figure 10. The circuit can be described as two charge island circuits (Goteti and Hamilton, 2018) coupled together, with charge on one of the islands affecting the switching dynamics of the other. Therefore, the weight of the synapse can be switched between 0 and 1 using voltage pulse signals at *V*_w_. When an incoming voltage write pulse *V*_w_ is larger than critical voltage *V*_c_ of *Q*_0_, the junction switches to a resistive state allowing a charge of 2*e* to the capacitor *C*_1_. The value of capacitance *C*_1_ is chosen such that it can only hold a charge of 2*e* before the voltage at node 1 exceeds the critical voltage of junctions *Q*_2_ and *Q*_3_ and the total charge on *C*_1_ discharges through the output current *I*_*b*1_. When the charge on capacitor *C*_1_ is zero, voltage pulse excitations from *V*_r_ do not induce transport of charge 2*e* across junction *Q*_1_ (Cheng et al., 2021), resulting in a weight of zero. When the charge on capacitor *C*_1_ is 2*e*, the resulting weight is 1, with one output spike per input pulse, as shown in Figures 11a–d. The weight can be decreased by increasing the capacitance of the capacitors *C*_1_ and *C*_2_ as shown in Figures 11e,f. When both the capacitances are doubled, the resulting weight becomes 0.5 with one output spike for every two input pulses.

**Figure 12:**
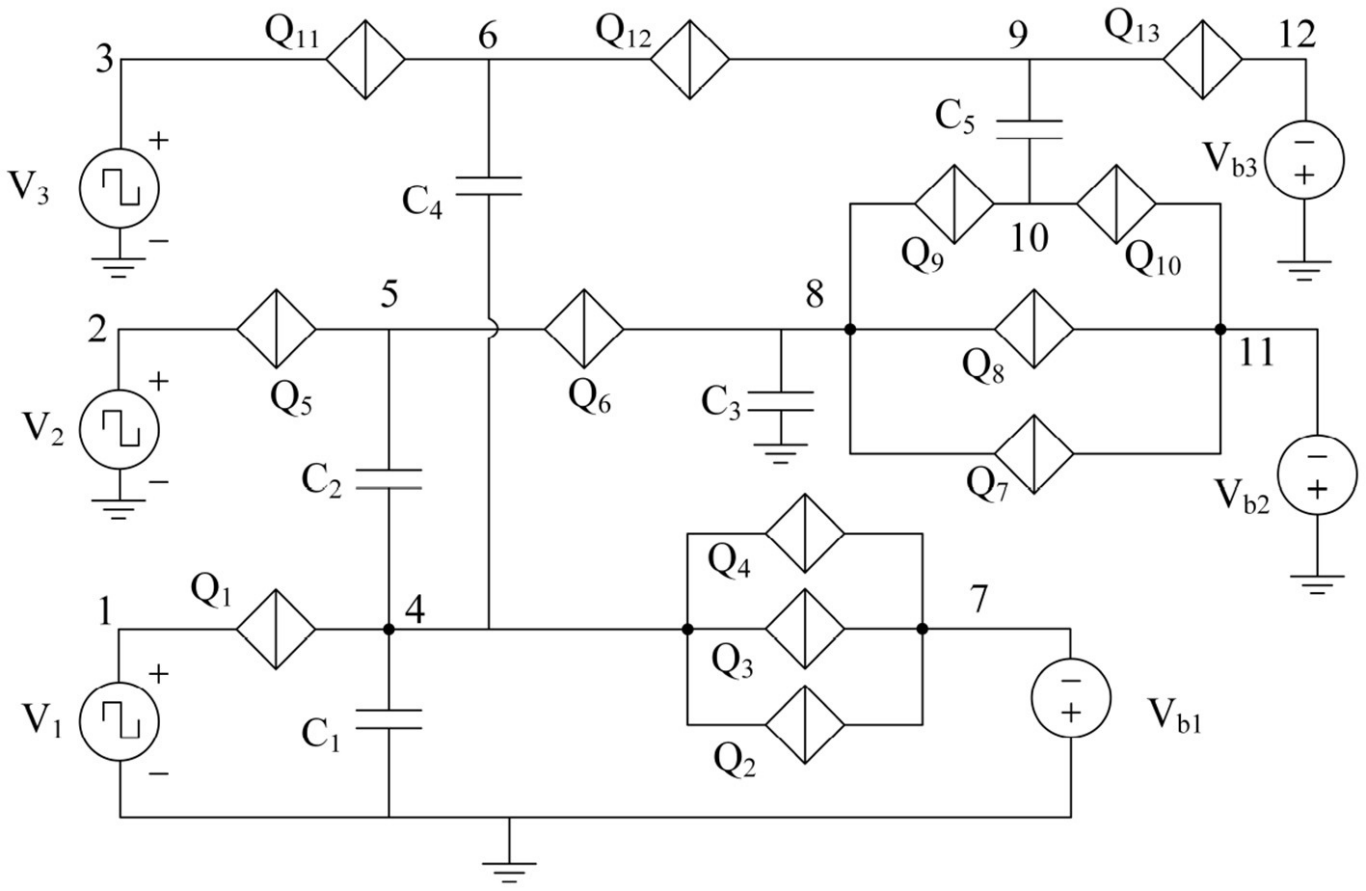
3 × 3 synapse network with 5 charge island capacitively coupled to each other. Capacitance values are given by: *C*_1_ = 6 fF, *C*_2_ = 3 fF, *C*_3_ = 8 fF, *C*_4_ = 2 fF, and *C*_5_ = 5 fF. Voltage pulse inputs are applied at *V*_1_, *V*_2_, and *V*_3_. Weights are programmed using bias voltages *V*_b1_, *V*_b2_ and *V*_b3_.

The binary synapse circuit described in Figures 10, 11 establishes that the weight depends on the capacitance values as well as the charge on the capacitors at any instant. Therefore, a multi-weight synapse network can be constructed by similarly coupling several charge islands of different capacitance values and corresponding QPSJ parameters. An example 3 × 3 network with 5 charge islands capacitively coupled to each other is shown in Figure 12. While the values of circuit parameters determine the weights achieved for different input signals, the actual choice of parameters is not crucial to demonstrate the properties of the 3 × 3 network. Additionally, the input junctions in the network are excited using voltage pulses of constant (i.e., not variable) amplitude. Each of these voltage pulses induce a charge of 2*e* into the network, and therefore represent quantized charge current spikes from the input neurons. The critical voltages of the junctions labeled from *Q*_1_ to *Q*_13_ are randomly chosen to exist between the range of 0.2–1.2 mV. The capacitance values chosen for simulations are given as: *C*_1_ = 6, *C*_2_ = 3, *C*_3_ = 8, *C*_4_ = 2, and *C*_5_ = 5 fF. The charge capacity of a charge island is given by $\frac{CV_{\text{c}}}{2e}$, where *C* is the capacitance of the capacitor on the island and *V*_c_ is the critical voltage of the smallest QPSJ in the island. Therefore, for the values chosen, each of the islands in the 3 × 3 network can accommodate a maximum charge ≥10. As the charge on one or more of the islands changes by 2*e* at any instant, the weights of all the synapses in the network are updated simultaneously. The coupled synapse network can be directly connected to the neuron circuits described in Figure 1 at each of its inputs and outputs to construct a fully connected neural network.

**Figure 13:**
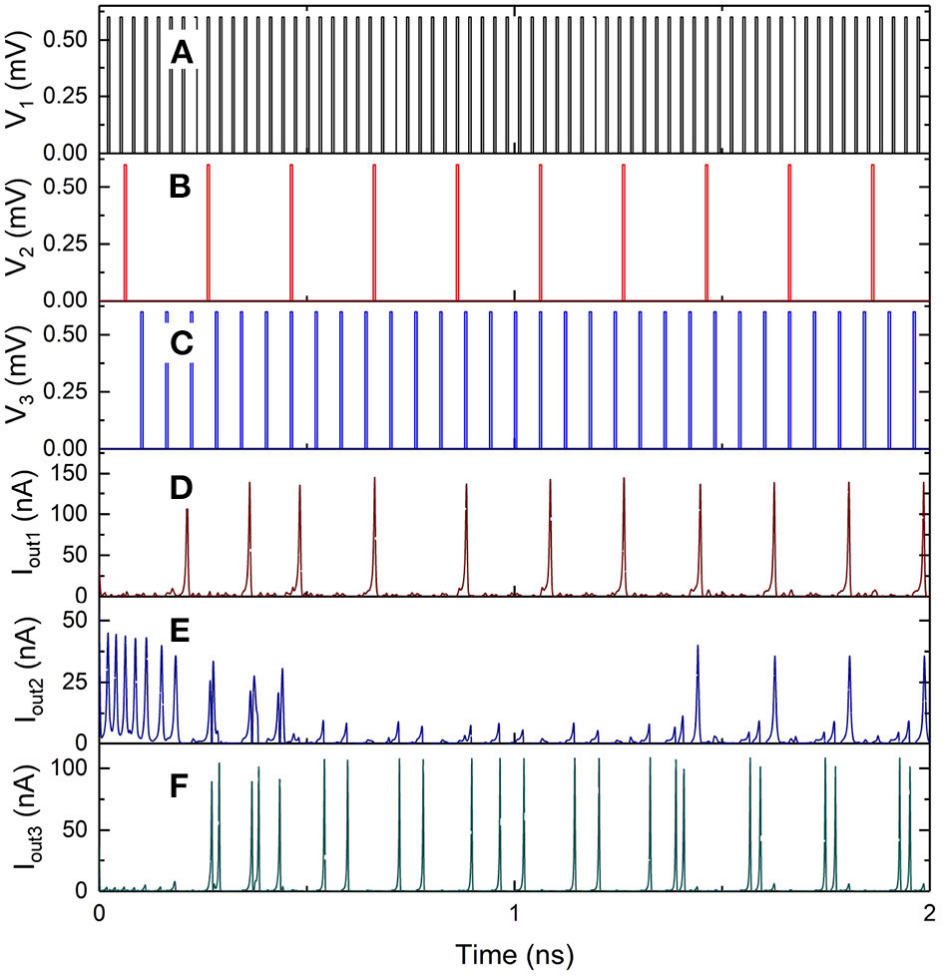
Simulation results of the 3 × 3 coupled synapse network shown in Figure 12. **(A)** Input voltage pulses of 0.6 mV with time period of 60 ps at *V*_1_. **(B)** Input voltage pulses of 0.6 mV with time period of 200 ps at *V*_2_. **(C)** Input voltage pulses 0f 0.6 mV with time period of 30 ps at *V*_3_. **(D)** Spiking current output at *I*_out1_. **(E)** Spiking current output at *I*_out2_. **(F)** Spiking current output at *I*_out3_.

The inputs *V*_1_, *V*_2_, and *V*_3_ to the 3 × 3 network shown in Figure 12 are excited using voltage pulses of constant amplitude of 0.7 mV but with different frequencies. The resulting output spiking signals are observed as a function of time, as shown in the simulation results in Figures 13A–F. The bias voltages *V*_b1_, *V*_b2_ and *V*_b3_ are constant at 0.7, 1.9, and 1.6 mV. With each voltage pulse at one of the inputs, the resulting charge on different islands is updated resulting in different dynamically varying output currents. These are observed as current pulse trains at the outputs, with each pulse comprising a charge 2*e*, with continuously changing time duration between consecutive spikes. The weight can be calculated as the fraction of the number of current pulses at the output with respect to the number of input pulses applied. Therefore, the variation of time duration between consecutive output spikes is evidence of dynamic updating of the synaptic weights in the network.

**Figure 14:**
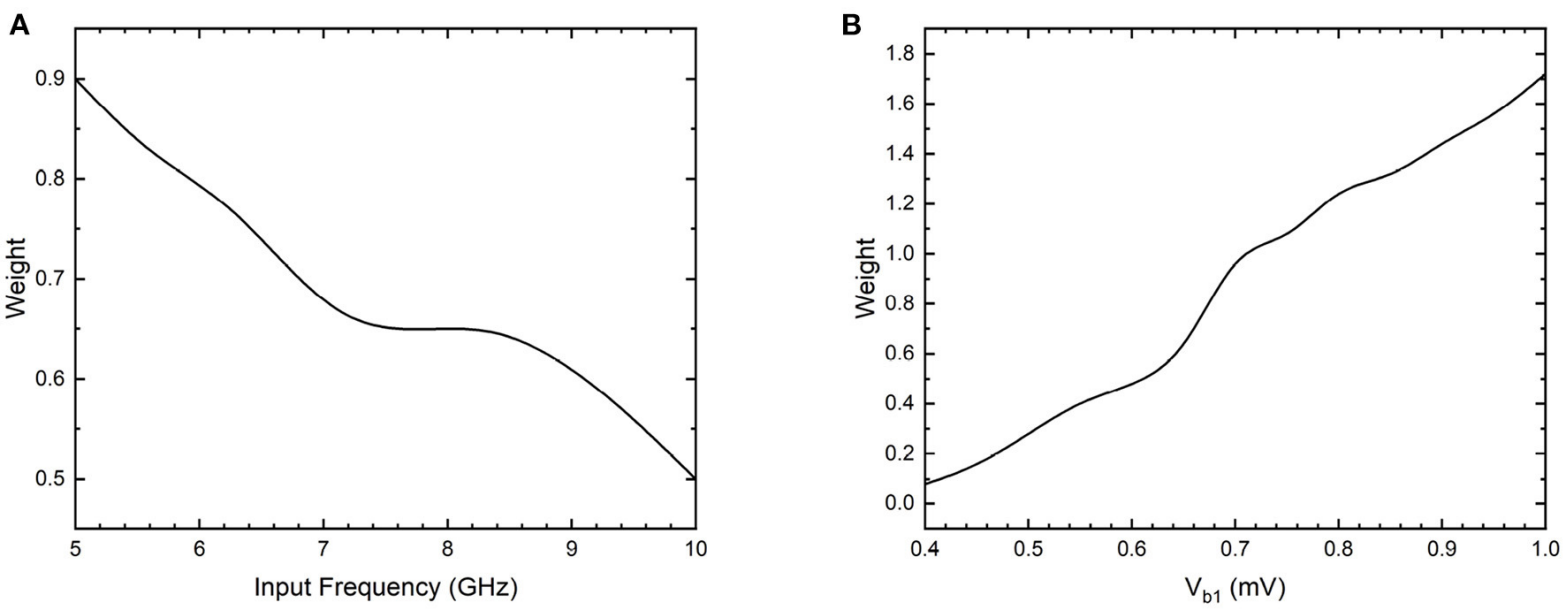
Simulation results of weight between an input-output node 1 in the 3 × 3 coupled synapse network shown in Figure 12, with an additional 100 kΩ resistor included parallel with *C*_1_. **(A)** Weight as a function of input frequency of *V*_1_ with constant bias voltages. **(B)** Weight as a function of bias voltage *V*_b1_ with input pulse voltage excitations of constant amplitude and frequency.

Additionally, the synapse networks can be configured to exhibit spike timing dependent plasticity with respect to input spiking signal timing by including resistors across the capacitors of the network, similar to LTD and LTP circuits in Figure 12. The resistors allow discharging of charge on the island with a fixed time constant for each node, thereby enabling STDP behavior with respect to input pulse frequency. When the bias voltage is constant, the synaptic weight between input *V*_1_ and output *I*_out1_ is dependent on the time period between input voltage pulses as shown in the Figure 14A. The weight decreases from 0.9 to 0.5 as the input frequency increases from 5 to 10 GHz. Similarly, the weight can also be configured using the bias voltage *V*_b1_ when the input pulse frequency is constant as shown in Figure 14B. During this operation, the weight increases from 0.1 to 1.7 as the bias voltage is increased from 0.4 to 1 mV. Therefore, the bias voltage and the input frequency have opposing effects on the weight of the synapse between input-output node. The bias voltage can either be coupled to the output signal in the form of a feedback loop, or can induce back-propagating charges with current flow in the opposite direction. The synapse then exhibits a spike timing and rate dependent plasticity with respect to both the input and the output signals.

The 3 × 3 network shown in Figure 12 can exhibit similar STDP learning behavior between weights of all of the 9 input-output connections shown by a weight matrix given as:


$$\left[\begin{array}{ccc} w_{11} & w_{12} & w_{13}\\ w_{21} & w_{22} & w_{23}\\ w_{31} & w_{32} & w_{33} \end{array}\right]$$

**Figure 15:**
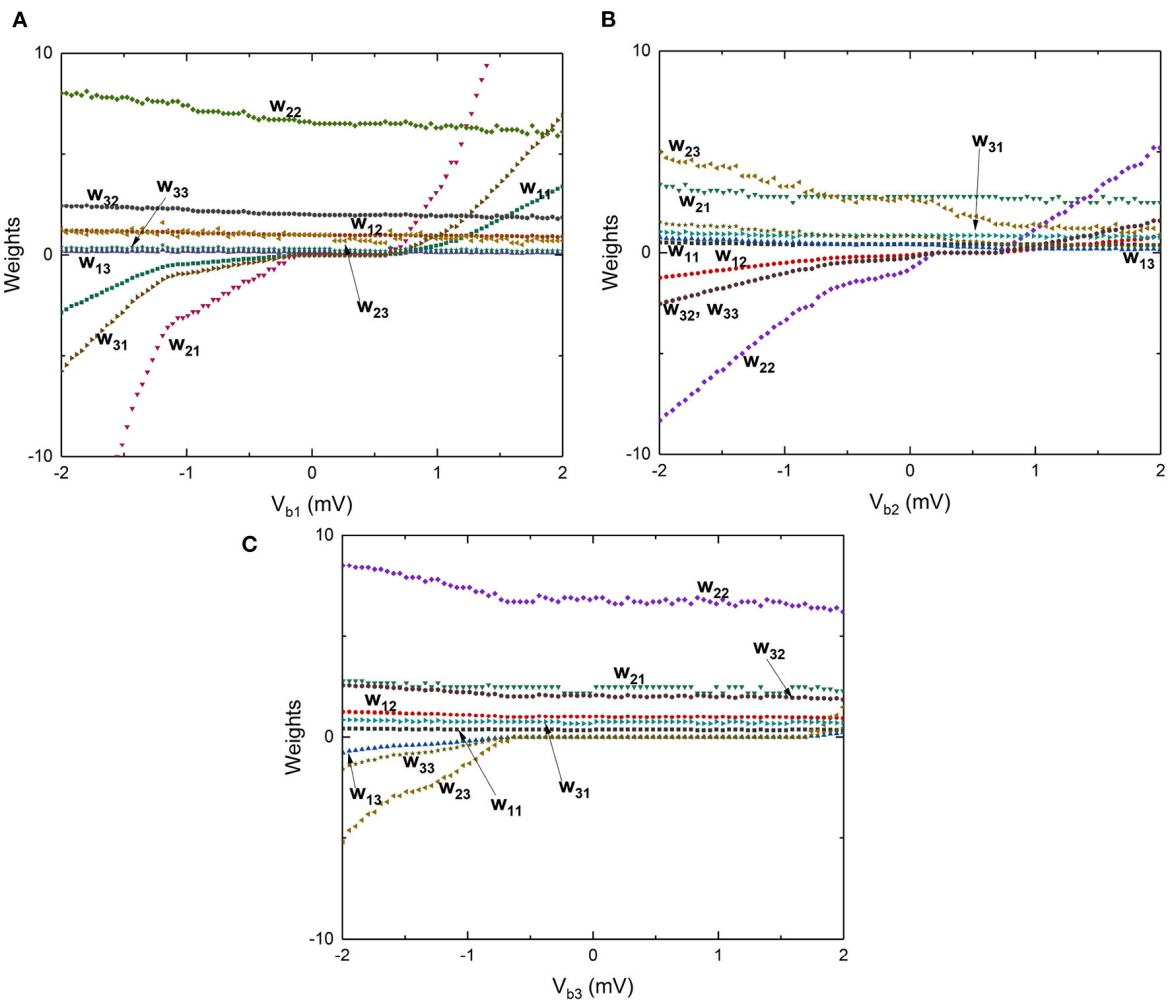
Large scale simulations performed by varying the bias voltages between -2 and 2 mV and measuring the weights in the matrix associated with the 3 × 3 network shown in Figure 12. **(A)** Weights vs. bias voltage *V*_b1_ with voltages *V*_b2_ and *V*_b3_ constant. **(B)** Weights vs. bias voltage *V*_b2_ with voltages *V*_b1_ and *V*_b3_ constant. **(C)** Weights vs. bias voltage *V*_b3_ with voltages *V*_b1_ and *V*_b2_ constant.

These weights are affected by any of the 3 input voltage pulse excitations and can also be programmed using the three bias voltage signals. To demonstrate this behavior, the circuit is simulated by independently changing the bias voltages between –2 and 2 mV and the weights between all the synapses in the 3 × 3 network, shown by the weight matrix, are plotted as a function of bias voltages in Figures 15A–F for bias voltages across *V*_b1_, *V*_b2_, and *V*_b3_. Voltage pulses of constant amplitude 0.6 mV are applied with different time periods of 30, 200, and 60 ps to inputs *V*_1_, *V*_2_, and *V*_3_, respectively. The results plotted in Figure 15 show that a large number of continuously varying weights can be realized in a synapse using only a few junctions by capacitively coupling different charge islands, with weights that can be controlled using bias voltages. Since the time-periods are constant at input excitations with constant bias voltages, STDP behavior is not explicitly observed. However, all the weights in the weight matrix are expected to be dynamically updated as a function of input frequency at each of the inputs, similar to the results shown in Figure 14A. Furthermore, different dynamical behaviors are observed in weights as shown in Figure 15. Weights *w*_11_, *w*_21_, and *w*_31_ show an increase in value with an increase in bias voltage *V*_b1_, with a plateau occurring between −−0.25 and 0.5 mV, where convergence in weights corresponding to a stable charge configuration on islands is observed. Similar behavior is observed in weights *w*_12_ *w*_22_, *w*_32_, and *w*_33_ with respect to bias voltage *V*_b2_, while weights between other input-output nodes continuously vary with bias voltages. These stable convergent regions are specific to the values of junction critical voltages and capacitance values chosen for the 3 × 3 network shown in Figure 12. Nevertheless, these results indicate that coupled synapse networks can be designed to demonstrate stable configurations that can be programmed using the bias voltages as desired by circuit designers and neural network programmers for specific neural network applications, thereby setting the stage for integration of coupled charge-island synapses into more complex neuromorphic circuits.

## 5. Discussion

In this section, we briefly discuss additional aspects of QPSJ operation temperatures, power or energy dissipation, and some of the expected design and experimental challenges related to QPSJ technology. Quantum phase-slip events have been previously observed at temperatures up to hundreds of mK (Aref et al., 2012) and recent experiments suggested coherent quantum phase-slips in NbN nanowires at temperatures up to 1.92 K (Constantino et al., 2018). We expect, and hope, that additional efforts in this area will allow materials, device structures and fabrication processes to be developed that will allow realization of coherent QPS at temperatures closer to 4 K. In the simulations we have performed, the QPSJ model was temperature independent, though once these dependencies are known, they can be included in more advanced QPSJ circuit models. As discussed in our previous papers (Cheng et al., 2018, 2021), QPSJ-based circuits, if fabricated properly, should have negligible static energy dissipation as the QPSJs are assumed to be in a Coulomb blockade condition when the voltage across them is less than their critical voltage. The primary energy dissipation during normal operation is assumed to be from the switching energy of each QPSJ that undergoes a switching event. Other than the energy dissipation within QPSJs, nominally only the resistors dissipate a small amount of energy, since the other circuit elements such as inductors and capacitors are assumed to be nearly ideal superconductive circuit elements. Since the currents used in these circuits are, in general, exceedingly small, the dissipation in the resistors is also small. For example, we have performed simulations to determine the energy dissipation in the circuit shown in Figure 4. The simulation results show that the energies dissipated at *V*_pre_, $\bar{V_{\text{post}}}$, *V*_b1_ and *V*_b2_ are 79.8, 134, 9.45, and 159 yJ, respectively, during each learning event.

In additional to biasing and impedance matching challenges, which were discussed in a previous section, device tolerance is also a concern for practical applications. For example, we have performed a small number of simulations to determine the tolerance for each QPSJ in the previously-presented multi-weight synaptic circuit (Cheng et al., 2021). The results indicated that the tolerance for identical parallel QPSJs is generally low (< 1%) while the tolerance for other QPSJs in the circuit is usually between a few to tens of percent. Therefore, device-to-device variation could affect the overall performance of the proposed circuit configurations, as it does in many electronic circuits. Once the fabrication technology is advanced to the point to realize relatively uniform, repeatable QPSJ devices, it will be important to optimize circuit designs, with the tolerances taken into account.

## 6. Conclusion

We have reviewed QPSJ-based superconducting neuromorphic circuits such as neurons and synapses and we have introduced new designs that enable STDP learning behavior. These circuits operate with spiking inputs and produce equivalent spiking outputs with each spike or current pulse comprised of a quantized charge 2*e*. The circuits for various neuromorphic network elements such as integrate-and-fire neurons, multi-weight synapses and fan-out mechanisms are discussed and demonstrated using SPICE circuit simulations. The simulation results indicate that artificial neural networks capable of learning through spike timing dependent plasticity can be constructed using QPSJs. STDP can be achieved in individual synapses through the LTD and LTP circuits presented, which allows deterministic control of weights and through a dynamic response to input excitations to the network. Alternatively, similar spike timing dependent plasticity can also be observed in the QPSJ-based coupled synapse network as demonstrated in 3 × 3 synapse network discussed in section 4. While these networks do not allow deterministic control of all the network parameters, neural networks can be constructed using this approach that may be useful to achieve spike-timing and rate dependent plasticity. In summary, QPSJs present a promising hardware platform to realize power efficient, high-speed spiking neural networks that are capable of both supervised and unsupervised learning.

## Data Availability Statement

The raw data supporting the conclusions of this article will be made available by the authors, without undue reservation.

## Author Contributions

RC created circuit designs and performed simulations for the circuits presented in sections 2 and 3. UG created circuit designs and performed simulations for the circuits presented in sections 2 and 4. HW, KK, and LO participated in discussions and contributed to manuscript preparation. MH led the project, participated in circuit design, simulation, analysis, led discussions, and manuscript preparation. All authors participated in manuscript preparation and revision processes.

## Funding

Funding and computing resources for this work performed was provided by Alabama Micro/Nano Science and Technology Center (AMNSTC) at Auburn University.

## Conflict of Interest

The authors declare that the research was conducted in the absence of any commercial or financial relationships that could be construed as a potential conflict of interest.

## Publisher's Note

All claims expressed in this article are solely those of the authors and do not necessarily represent those of their affiliated organizations, or those of the publisher, the editors and the reviewers. Any product that may be evaluated in this article, or claim that may be made by its manufacturer, is not guaranteed or endorsed by the publisher.
