# Supplementary material for: Toward Learning in Neuromorphic Circuits Based on Quantum Phase Slip Junctions
Source: Front Neurosci. 2021 Nov 8;15:765883. doi: 10.3389/fnins.2021.765883 (PMC8606638; doi:10.3389/fnins.2021.765883)
Supplement: Supplementary file 1 [file Data_Sheet_1.PDF]

Supplementary Material

1 SUPPLEMENTARY TABLES AND FIGURES

Most of the simulation parameters for simulation results shown in the paper are presented here.

Component	Value	Comment
V_{in}	0.8 mV	Input voltage
Q_1-Q_{10}	0.7 mV	Critical voltage
C	15 fF	Capacitance
V_b	1 mV	Bias voltage
R_b	9 kΩ	Resistance

Table S1. Device parameters for the simulation shown in Figure 1.

Component	Value	Comment
V_r	0.54 mV	Read voltage
V_w	0.7 mV	Write voltage
Q_0	0.3 mV	Critical voltage
Q_1	0.5 mV	Critical voltage
Q_2	0.52 mV	Critical voltage
Q_3	0.54 mV	Critical voltage
Q_4	0.31 mV	Critical voltage
C_1	9.2 fF	Capacitance
C_2	1.2 fF	Capacitance
V_{b1}	0.5 mV	Bias voltage

Table S2. Device parameters for the simulation shown in Figure 2.

Component	Value	Comment
Q_1-Q_{10}	0.5 mV	Critical voltage
L_1, L_2	0.1 nH	Inductance
L_3	0.01 nH	Inductance
V_b	0.5 mV	Bias voltage
I_b	70 μA	Bias current
J_1	40 μA	Critical current
J_2	50 μA	Critical current

Table S3. Device parameters for the simulation shown in Figure 3.

Component	Value	Comment
$\underline{V_{pre}}$	0.54 mV	presynaptic voltage
$\overline{V_{post}}$	0.95 mV	postsynaptic voltage
Q_0	0.75 mV	Critical voltage
Q_1	0.56 mV	Critical voltage
Q_2	0.31 mV	Critical voltage
C_1	9.2 fF	Capacitance
C_2	1.2 fF	Capacitance
R_1	10/20/30/40 kΩ	Resistance
V_{b1}	0.03 mV	Bias voltage
V_{b2}	0.5 mV	Bias voltage

Table S4. Device parameters for the simulation shown in Figure 5.

Component	Value	Comment
$\underline{V_{pre}}$	0.54 mV	presynaptic voltage
$\overline{V_{post}}$	0.95 mV	postsynaptic voltage
Q_0	0.75 mV	Critical voltage
Q_1	0.55 mV	Critical voltage
Q_2	0.3 mV	Critical voltage
Q_3	2 mV	Critical voltage
Q_4	0.54 mV	Critical voltage
Q_5	0.52 mV	Critical voltage
Q_6	0.5 mV	Critical voltage
Q_7	0.34 mV	Critical voltage
C_1	9.2 fF	Capacitance
C_2	1.2 fF	Capacitance
C_3	9.2 fF	Capacitance
C_4	1.2 fF	Capacitance
R_1	10 kΩ	Resistance
V_{b1}	0.03 mV	Bias voltage
V_{b2}	0.77 mV	Bias voltage
V_{b3}	0.53 mV	Bias voltage

Table S5. Device parameters for the simulation shown in Figure 6.

Component	Value	Comment
V_{pre}	0.78 mV	presynaptic voltage
$\overline{V_{pre}}$	0.54 mV	presynaptic voltage
V_{post}	0.51 mV	postsynaptic voltage
Q_0	0.4 mV	Critical voltage
Q_1	0.5 mV	Critical voltage
Q_2	1 mV	Critical voltage
Q_3	1 mV	Critical voltage
Q_4	0.58 mV	Critical voltage
Q_5	2 mV	Critical voltage
Q_6	1.04 mV	Critical voltage
Q_7	1.02 mV	Critical voltage
Q_8	1 mV	Critical voltage
Q_9	0.28 mV	Critical voltage
C_1	9 fF	Capacitance
C_2	1 fF	Capacitance
C_3	9.2 fF	Capacitance
C_4	2 fF	Capacitance
R_1	10 kΩ	Resistance
R_2	10 kΩ	Resistance
V_{b1}	0.05 mV	Bias voltage
V_{b2}	0.2 mV	Bias voltage
V_{b3}	1.1 mV	Bias voltage
V_{b4}	1.01 mV	Bias voltage
V_{b5}	0.6 mV	Bias voltage

Table S6. Device parameters for the simulation shown in Figure 7.

Component	Value	Comment
V_{pre}	0.78 mV	presynaptic voltage
V_{post}	0.51 mV	postsynaptic voltage
Q_0	0.8 mV	Critical voltage
Q_1	0.95 mV	Critical voltage
Q_2	0.95 mV	Critical voltage
Q_3	0.36 mV	Critical voltage
Q_4	0.5 mV	Critical voltage
Q_5	1 mV	Critical voltage
Q_6	1 mV	Critical voltage
Q_7	0.58 mV	Critical voltage
Q_8	2 mV	Critical voltage
Q_9	1.04 mV	Critical voltage
Q_{10}	1.02 mV	Critical voltage
Q_{11}	1 mV	Critical voltage
Q_{12}	0.28 mV	Critical voltage
C_1	1 fF	Capacitance
C_2	9 fF	Capacitance
C_3	1 fF	Capacitance
C_4	9.2 fF	Capacitance
C_5	2 fF	Capacitance
R_1	10 kΩ	Resistance
R_2	10 kΩ	Resistance
R_3	10 kΩ	Resistance
V_{b1}	0.2 mV	Bias voltage
V_{b2}	1.1 mV	Bias voltage
V_{b3}	0.05 mV	Bias voltage
V_{b4}	0.2 mV	Bias voltage
V_{b5}	1.1 mV	Bias voltage
V_{b6}	1.01 mV	Bias voltage
V_{b7}	0.6 mV	Bias voltage

Table S7. Device parameters for the simulation shown in Figure 8.

Component	Value	Comment
V_{pre}	1.07 mV	presynaptic voltage
V_{post}	0.51 mV	postsynaptic voltage
$\overline{V_{post}}$	0.51 mV	postsynaptic voltage
Q_0	0.8 mV	Critical voltage
Q_1	0.95 mV	Critical voltage
Q_2	0.95 mV	Critical voltage
Q_3	0.36 mV	Critical voltage
Q_4	0.5 mV	Critical voltage
Q_5	1 mV	Critical voltage
Q_6	1 mV	Critical voltage
Q_7	0.46 mV	Critical voltage
Q_8	2 mV	Critical voltage
Q_9	0.75 mV	Critical voltage
Q_{10}	0.55 mV	Critical voltage
Q_{11}	0.3 mV	Critical voltage
Q_{12}	1.37 mV	Critical voltage
Q_{13}	1.35 mV	Critical voltage
Q_{14}	1.33 mV	Critical voltage
Q_{15}	0.28 mV	Critical voltage
C_1	1 fF	Capacitance
C_2	9 fF	Capacitance
C_3	1 fF	Capacitance
C_4	9.2 fF	Capacitance
C_5	1.2 fF	Capacitance
C_6	9.2 fF	Capacitance
C_7	2 fF	Capacitance
R_1	10 kΩ	Resistance
R_2	10 kΩ	Resistance
R_3	20 kΩ	Resistance
R_4	10 kΩ	Resistance
R_5	20 kΩ	Resistance
V_{b1}	0.2 mV	Bias voltage
V_{b2}	1.1 mV	Bias voltage
V_{b3}	0.05 mV	Bias voltage
V_{b4}	0.2 mV	Bias voltage
V_{b5}	1.1 mV	Bias voltage
V_{b6}	0.89 mV	Bias voltage
V_{b7}	0.46 mV	Bias voltage
V_{b8}	0.3 mV	Bias voltage
V_{b9}	0.6 mV	Bias voltage

Table S8. Device parameters for the simulation shown in Figure 10.
